# Discovering the sensory, emotional, and interactive experiences of a place

**DOI:** 10.3389/fpsyg.2024.1303397

**Published:** 2024-06-05

**Authors:** Luis Alfonso de la Fuente Suárez

**Affiliations:** Architecture School, Autonomous University of Nuevo Leon, San Nicolás de los Garza, Mexico

**Keywords:** environmental experience, place preference, emotions, sensory experience, architecture

## Abstract

This article proposes a data collection technique for describing experiences of a built environment. Besides the experiences of the visual and physical aspects of the place, this technique helps describe the sensory, bodily, emotional, interactive, and social experiences occurring during the human-environment encounter. The enabling technique presented is called Reactions and Actions Description Survey (RADES). It employs 120 images depicting people going through different situations involving all the senses, showing expressions related to positive and negative emotions, and realizing varied activities. Forty-five participants visited the esplanade in the exterior of a historic building called Obispado. The case study is located on a hill and is a scenic viewpoint of Monterrey, Mexico. The participants answered the RADES and the Environmental Description Survey (ENVIDES), which focuses on describing the qualities of the place and the appraisals with which it is experienced. The comments about the experiences of the place obtained through both surveys were grouped into 133 categories. Qualitative and quantitative data about the experiences of the place were obtained through both techniques. A quantitative analysis of the data was realized since the participants not only described their experiences with words but also indicated numerically the intensity of such experiences. Spearman correlations between the experiences were calculated, and a general map of the experiences of the place was created through multidimensional scaling analysis (MDS). The study revealed the connections between the elements and qualities of the site and the views with specific positive and negative experiences occurring during the visit. Furthermore, MDS allowed the discovery of 10 dimensions of environmental experience—pleasure/displeasure, high/low arousal, dominance/submissiveness, knowing/inhabiting, environment/self, higher/lower cognition, spatiality/materiality, states/processes, natural/built, and visual/sensory. The presented techniques and the findings obtained through them can assist architects in recognizing valuable environmental features for the design of livable spaces.

## 1 Introduction

Places are the centers of human experiences of the world, which are characterized by their physical setting, ongoing activities, and meanings (Relph, 1976). This research intends to study a broad set of human experiences in a particular place, from bodily sensations, such as paying attention to breathing, to the aesthetic experience of the landscape or architecture. Due to the multifarious possibilities of subjective experiences in a place, the following introduction includes topics about experiences from several disciplines and approaches. The introduction is divided into five subchapters in which definitions and distinct types of human experience are presented. The emotional, sensory, and interactive experiences of places have been identified as three general types of experience that go beyond the visual and material qualities of the environment and have been inquired in existing research. Sections discussing these experiences are included in the introduction to align with the focus of the technique presented in this article, which aims to study such experiences. Existing research utilizing techniques for investigating people's experiences of places are referenced throughout the introduction for comparison with the proposed technique.

The introduction starts with the subchapter about emotions since they are considered “an essential building block of consciousness” (Nummenmaa et al., 2018, p. 9198). In this section, several models and theories of emotions are presented. The second subchapter treats the sensory and bodily experiences and focuses on how the stimuli from the environment or the body are felt by a human being. The third section concerns the types of activities in which people are involved while being in a place. The existing definitions of consciousness, experience, and experience of place in general are treated in the fourth subchapter. The last section introduces the topic of the applicability of understanding human experiences of places in the design of such places. The approach followed in this study and the presented technique is expected to be better understood after this introduction, even though such an approach does not strictly follow the theories to be exposed here.

### 1.1 Emotions and emotional experiences

According to Russell and Mehrabian (1977), emotional states can be defined through three dimensions—pleasure, arousal, and dominance (PAD). The pleasure-displeasure dimension differentiates the positive (tranquility and excitement) and the negative (anger and boredom) affective states (Mehrabian, 1996). Pleasure-displeasure relates to the evaluation dimension proposed by Osgood et al. (1957). Good-bad and beautiful-ugly have high loadings in the semantic differential factor of evaluation. The second dimension, arousal, encompasses the level of physical or mental activity (Mehrabian, 1996). In this manner, relaxation is a low arousal state, while concentration is a high arousal state. Arousal is somewhat related to the activity dimension (Osgood et al., 1957), in which fast-slow and passive-active have high loadings. The Affect Grid (Russell et al., 1989), which includes the dimensions of pleasure-displeasure and arousal-sleepiness, was employed to study the emotional experiences of architecture in a study by Gregorians et al. (2022).

Dominance-submissiveness, the third dimension of the PAD model, is the feeling of controlling and influencing, or, on the contrary, being controlled and influenced by a situation. Anger and power are located in the dominance extreme, while on the submissiveness side reside anxiety and fear (Mehrabian, 1996). Dominance may relate negatively to the potency dimension that Osgood et al. (1957) described. Large-small and strong-weak exemplify the potency dimension. A weak and small stimulus (low potency) may elicit an emotion of higher dominance (Mehrabian, 1996). It is important to remark that PAD focuses on the responses or feelings of people, while the EPA model is about the experiences with the stimuli (Bakker et al., 2014). It is also relevant to highlight that evaluation, activity, and potency (EAP) and pleasure, arousal, and dominance (PAD) are abstract dimensions that may be discovered through a multidimensional scaling but are not necessarily part of people's subjective experiences (Shaver et al., 1987). Before PAD and EAP, Wundt (1912) also developed a three-dimensional model of emotions. His model included the opposite feelings of pleasure/pain, strain/relaxation, and excitation/quiescence.

Apart from the dimensional theories of emotion, the categorical theories propose the existence of a set of basic emotions (Fujimura et al., 2012). There are 10 fundamental emotions, as stated by Izard (1977): interest, joy, surprise, distress, anger, disgust, contempt, fear, shame, and guilt. Each of them “has unique motivational properties of crucial importance to the individual and the species, and each adds its own special quality to consciousness as it mobilizes energy for physical or cognitive adventure.” (Izard, 1977, p. 83). Nevertheless, other authors propose more reduced sets of basic emotions (Shaver et al., 1987). There is no agreement on the number of basic dimensions since the definition of emotion is still undefined (Crivelli and Fridlund, 2019). According to Ekman (1992), there are families of basic emotions, such as anger, fear, disgust, and sadness, which have specific facial expressions, physiology, and particular events in which they occur. The same author presents several characteristics distinguishing emotions from other affective states; for instance, emotions begin quickly, have a brief duration, and are unbidden, i.e., they happen to us, and we do not choose them.

From another approach, emotions emerge from the person's evaluations (appraisals) of the environmental changes that result relevant to wellbeing, according to appraisal theory (Ellsworth, 2013). An event's perceived outcome determines the emotion; that is a central principle of appraisal theory (Scherer, 2001). Ellsworth (2013, p. 126) specified that “appraisal change is an emotional change,” and the physiological responses, expressions, subjective experiences, and action tendencies correlate with the appraisals. Similarly, Cochrane (2009) indicates that emotions may manifest in the following forms: experienced feelings, appraisals, expressive behavior, actions, language, and physiology.

The checklist created by Davitz (1969) is one of the most extensive sets of descriptions of physical sensations, thoughts, and behaviors used to study emotional experiences. The checklist helps report how it is to go through a specific situation or event. The 556 items on the checklist include statements describing the following aspects:

a) Physical sensations: “I'm thirsty”; “I feel tired, sleepy”; “I become conscious of my breathing.”
b) Perception and relation to the external situation: “everything seems quiet”; “I have a sense of strangeness, unreality, as if I'm temporarily in another world”; “everything seems in place, ordered, organized.”
c) Cognitive events: “I feel disoriented”; “I am able to think clearly, understand everything”; “I become introspective, turn inwards.”
d) One's relation to others: “I want to talk to someone”; “I want to be with friends”; “a sense of belonging with others.”
e) One's self: “a sense of being very integrated and at ease with myself, in harmony with myself”; “I feel safe and secure”; “I feel I can really be myself.”
f) Impulses to behave or control behavior, and expressive behaviors: “I don't want to move; I just want to stay motionless, immobile”; “I have a sense of being free, uninhibited, open, no longer blocked. I feel uninhibited and spontaneous—anything goes”; “I feel like slouching, just being limp, completely relaxed.”
g) Formal aspects of the experience: “the feeling seems to be all over, nowhere special, just not localized”; “the feeling goes slowly”; “the feeling seems to be mostly in my muscles.”

Fifty participants were asked to use the checklist to describe 50 emotions. A Dictionary of Emotional Meaning was created by Davitz (1969) based on these descriptions. In such a dictionary, the description of the emotional experiences is presented through the checklist items. In the case of awe, the following items are the ones with the highest percentages and presumably are the ones that better describe the experience: I'm excited in a calm way (66%), I feel wide awake (50%), and there is a renewed appreciation of life (50%).

### 1.2 Feelings, sensory, and bodily experiences

The term feeling is employed by Nummenmaa et al. (2018) to refer to a person's subjectively accessible phenomenological state. Therefore, specific feelings accompany emotions, cognitive states, and bodily experiences. The similarity of 100 subjective feelings was measured, and the feelings were mapped to create a Feeling Space (Nummenmaa et al., 2018). According to this study, most feelings are experienced as emotional, and the resulting Feeling Space is organized in a way in which pleasant and unpleasant feelings are clearly separated. Furthermore, five clusters of feelings were found in the Feeling Space: (a) positive emotions: e.g., relaxation, togetherness; (b) negative emotions: tiredness, disappointment; (c) cognitive processes: being conscious, thinking; (d) somatic states and illnesses: feeling pain, dizziness; and (e) homeostatic states: hunger, breathing. In the same study, somatic states and illnesses presented a high bodily salience (how much the feeling was felt in the body), while cognitive processes had a low bodily salience.

The body allows us to move; it is the instrument through which we dwell, experience, and respond to space and is an experienced space itself (Bollnow, 2020). The body is not a subject nor an object but a point in between (Bollnow, 2020). The distinction between bodily sensations and bodily feelings is remarked on by Armstrong (1962). He suggests that bodily sensations, such as pains and itches, are always located in a more or less extensive body part. Meanwhile, bodily feelings are not confined to a specific part of the body, such as feeling tired or sleepy. Another important distinction made by Armstrong (1962) is the one between what he calls transitive and intransitive sensations. On the one hand, transitive sensations can exist without someone experiencing them, such as warmth or pressure, but they may also be experienced, such as in a sensation of warmth or pressure. Generally speaking, transitive sensations are about the experience of an external object in contact with our body. On the other hand, intransitive sensations cannot exist without a sentient being; that is the case of pain, which is always a sensation of pain. Intransitive sensations are about bodily states, according to the author.

A sensory experience is not merely what occurs while being in an environment offering certain stimuli (light, sound, heat, etc.) but the experience that arises when a human being focuses on how such stimuli are felt through the senses and body (de la Fuente Suárez, 2016, 2019). Nevertheless, most times, sensations are not attended to and are only a sign of an external object, which gives the name to the sensation, such as the sensation of a flavor, sound, motion, or heat (Armstrong, 1962; Reid, 2004). Only when a sensation is too pleasant or unpleasant is it attended to, and a name is assigned to it, as happens with the types of pains and the sensations of hunger or sickness (Reid, 2004).

Regarding the techniques to inquire the sensory experiences of places, some authors employ visual analog scales with pairs of adjectives to specify the qualities of the stimuli (Malnar and Vodvarka, 2004; Mace, 2014), e.g., signal (foreground sound) vs. keynote (background sound). Meanwhile, other techniques allow participants to select adjectives from a list to describe the stimuli obtained through each of the senses. Rough/smooth, hard/soft, and warm/cold are examples of tactile descriptors included in the sensory notation created by Lucas and Romice (2010). Moreover, this notation allows us to indicate the priority rank of each sense and the corroboration between qualities acquired through different senses.

According to Baars (1997, p. 369), “Nothing in human experience is as rich and full of subtle details as the sensory world.” Nevertheless, multiple theoretical works have stressed that contemporary built environments lack such sensory richness and that sight is the privileged sense (Neutra, 1954; Pallasmaa, 2012; Plummer, 2016). Erwine (2017) indicates that the controlled homogeneity and predictability of the sensory qualities of modern built environments give people comfort but deprive them of delight. Many authors have based their theories about architectural phenomenology on the search for multisensoriality of inhabitable spaces (Erwine, 2017). Nevertheless, more empirical research about sensory experiences of built environments beyond the visual ones is needed (Coburn et al., 2017). Many studies on the sensory experiences of places have focused on a single sensory domain (Spence, 2020). How stimuli obtained by distinct senses interact during an experience is little known (Spence, 2022). The interrelations between the experiences of a place, which are of prime importance in this study, can be better understood with the following comment by Erwine (2017, p. 31): “…the tinge of rotting seaweed at the seashore—does it lessen or heighten the contrasting freshness of the sea breeze?”

### 1.3 Social and interactive experiences of a place

The lifeworld (van Manen, 1990) is the world as it is experienced and lived in quotidian situations. The author identifies four interrelated aspects or existentials, which are part of everybody's lifeworlds and may be used in the study of human experiences—lived space, lived body, lived time, and lived human relation. Lived space is about how a place or space makes people feel and how certain activities or experiences are connected to specific places. Lived body or corporeality refers to the experiences of being bodily in the world. Lived time refers to the experience of time had during a situation and also a person's past, present, and future life. Lastly, lived human relation (relationality or communality) is the bond we have with people, the social relations in which “we transcend our selves” (van Manen, 1990, p. 105). A distinction between unfocused and focused interactions between people is made by Goffman (1963). The mere presence of people in the same place produces an unfocused interaction; they are aware of each other but do not talk. Meanwhile, in a focused interaction, people cooperate or communicate. The latter type of interaction is commonly considered a social interaction. The social interaction scale (SIS) created by Chen et al. (2023) employs three levels to measure socialization in urban parks: (1) a person who is alone in the place; (2) people in a group who are more focused on the activity than on the partners; and (3) a group of people engaged in activities together. According to Chen et al. (2023), objectively measuring the level of social interaction in places allows one to discover connections between socialization and the qualities of such places. The study of socialization is a relevant area of research since designers create environments that positively influence social interactions, such as friendship formation, group membership, and communications (Deasy and Lasswell, 1985).

Besides the experiences of socialization, there are interactive experiences of the environment involving the transformation of the elements of a place or the realization of activities in it (de la Fuente Suárez, 2013). The sense of agency is the awareness of our acts and that we are the ones who cause them (Gillihan and Farah, 2005). We are conscious of the external environment, and our minds guide our behavior (Revonsuo, 2010). The experience of a place is not a passive and static look but an interaction between a person and the environment (de la Fuente Suárez, 2013). While moving through a place, a person experiences his or her movement and simultaneously experiences a change in the external world (Gibson, 1986; Kalin and Yilmaz, 2012). The aesthetic triad by Coburn et al. (2017) presents three systems that generate aesthetic experiences of architecture. In this model, the sensory-motor system relates to both (a) the stimulation received through the senses and (b) peoples' movement, motivation, and responses, such as approach or avoidance (Coburn et al., 2017, 2020). The other two systems of the triad are emotion-valuation and knowledge-meaning.

One common type of human interaction with the environment occurs when a person wants to know a place. Exploration is “the choice of actions with the goal of obtaining information,” according to Gottlieb et al. (2013, p. 586); therefore, exploratory acts do not intend to transform the external world but to gain knowledge. Humans are constantly looking for new information in their environment (which relates to curiosity), and such information-seeking may have no other benefit than learning itself (Gottlieb et al., 2013). Some environments may elicit a higher exploratory behavior and curiosity than others. The sense of mystery described by Kaplan (1987) is about places configured in a way that induces exploration by revealing some information while hiding other information that can be predicted or inferred.

### 1.4 Consciousness, experience, and the description of experience

According to Mallgrave (2007, p. 166), “Consciousness entails the process by which we come to be aware of ourselves, the world, and the constancy of this relationship over time.” Similarly, Laughlin and Rock (2013) state that consciousness is the awareness of the external and the internal spaces. Besides the experiences of the external world (the things in the environment) and the internal world (ideas and inner sights), Dennett (1991) proposes the category of experiences of affect, which includes emotional and sensory experiences.

It is important to distinguish here between the concepts of consciousness and experience. Nevertheless, a warning should be made since a “universally accepted definition” of consciousness does not yet exist (Laughlin and Rock, 2013, p. 204). Consciousness is what allows the existence of subjective experiences, but consciousness is not the same as its contents, that is, the experiences (Revonsuo, 2010). Additionally, consciousness should not be confused with perception since there are conscious contents that are not perceptual, such as mental images or intentions (Baars, 1997).

As stated by Block (1995, p. 230), “The totality of the experiential properties of a state are ‘what it is like' to have it.” A concept that is synonymous with experience is that of phenomenon. Varela and Shear (1999, p. 3) indicate that a phenomenon is “an appearance and therefore something relational. It is what something is for something else; it is a being for by opposition to a being in itself independently of its apprehension by another entity.” The modes of appearance of a phenomenon are the distinct ways in which people can experience something (Bevan, 2014).

Revonsuo (2010) lists the following possible types of experiences that may be part of the subjective psychological reality of a person: the perceptual experiences obtained through the distinct senses, the emotional and bodily experiences, the desires, thoughts, memories, mental images, and inner speech. In a similar manner, Saoji and Bahadure (2012, p. 904) remark on the broad range of aspects included in an experience, in particular, that of an architecture work: “Experience is more than an observation, more than knowledge of a space or object and more than a feeling. It is description of the complete, encompassing influence of a space, object, or person on the user through mind, perception, and senses.” The multiple facets present during an architectural experience can be summarized with the sensorimotor, knowledge-meaning, and emotion-valuation systems proposed by Coburn et al. (2017).

Since subjective experience conveys what it is like to have it, and it is not about the objects themselves but how they appear to someone, first-person reports are the only way to study human experiences. A researcher of experiences has access to the description of experiences given by the participants but not to the experiences themselves (Petitmengin and Bitbol, 2009). Contrary to behavior, experiences cannot be observed from an outside perspective (Barrett et al., 2007; Revonsuo, 2010). Merleau-Ponty (2002, p. vii) indicates that phenomenology “tries to give a direct description of our experience as it is, without taking account of its psychological origin and the causal explanations which the scientist, the historian or the sociologist may be able to provide.” It is important to remark on the difference between Phenomenology with upper case and phenomenology with lower case (Dennett, 1991). The first refers to the philosophical schools, while the second is about the conscious experiences themselves.

The type of introspection that is useful for the empirical study of experiences and is employed in the present study is descriptive introspection (Revonsuo, 2010). In this introspection, a participant describes his or her experiences as they appeared without trying to give an interpretation or explanation of the phenomena. Few qualitative studies focus on people's experiences in real architectural environments. One such study by Kuliga et al. (2013) identifies the positive and negative experiences of the Seattle Public Library through semi-structured interviews. Besides the positive aesthetic experience generated by the building, the problems with navigation through the distinct floors caused the building to be experienced as a maze, according to the participants. Another mention-worthy study is by Weisen (2021), who conducted micro phenomenological interviews to explore the emotional, sensory, and existential experiences of visitors to the Kolumba Museum by Peter Zumthor. During the visit to the museum, several experiences related to an improved mood state and the enhancement of the sense of self occurred to the participants. Besides interviews, concurrent think-aloud protocols (de la Fuente Suárez, 2019, 2020) and enabling techniques in which comments are given by completing phrases (de la Fuente Suárez, 2022) have also been used to investigate subjective experiences of places and buildings.

In other respects, several data collection instruments employ figures of human bodies or faces to assess people's emotions under specific circumstances. Interesting cases of these instruments are the Self-Assessment Manikin (Bradley and Lang, 1994) and the Affective Slider (Betella and Verschure, 2016), both including pleasure and arousal dimensions. The Self-Assessment Manikin was used to rate the pleasure, arousal, and dominance of virtual architectural interiors (Banaei et al., 2017). Meanwhile, the Product Emotion Measurement Instrument (PrEmo) by Desmet (2004) employs animated human figures to evaluate seven pleasant and seven unpleasant emotions. It is important to remark that the techniques mentioned above are only centered on the emotional aspects of the experiences. Furthermore, these techniques are quantitative tools in which the participants cannot comment on their experiences.

### 1.5 Inquiring and designing experiences of places

Noticeably, many theorists and researchers focus on one aspect of experience while describing it. Some of them mainly focus on the affective or emotional aspect of the experience; others emphasize the sensory aspect of experiencing a place or the embodied encounter of that experience; few others are centered on the quotidian lived experience. Depending on the specific qualities of a place, some of the previous approaches may be more suitable than others to study the experiences of such a place. Nevertheless, the present study and the proposed technique pretend to encompass as many possible facets of the experience of a place without tending to emphasize any of them over the others.

Most designers have a set of simple ideas or assumptions about how people experience the environment, and they design places according to such assumptions (Temple, 2013). The importance of research on the experiences of places resides in how it opens up possibilities for design based on empirical data. As explained by Temple (2013), people's experiences are the primary measure of the efficacy of an architectural design. Therefore, designers must consider the “shape of the experience of architecture” and not just the “shape of the architecture” (Temple, 2014, p. 7). The generation of experiences in people through the design of places and architecture has been addressed by de la Fuente Suárez (2013) and Erwine (2017).

In a more specific manner, places can be designed to provide wellbeing to human beings. A brief review of existing research connecting the physical qualities of a place with positive outcomes in people is presented in the following lines. The model for creating healthy environments by Stoltz and Grahn (2021) is based on eight perceived sensory dimensions. Such dimensions compose four axes of opposing pairs—natural/cultural, cohesive/diverse, sheltered/open, and serene/social, in which the first dimension is a restorative quality, while the second is a more demanding one (Stoltz, 2022). The relevance of the model resides in its capability to characterize a broad range of environments with a reduced set of dimensions, which are vital for understanding human-environment relations.

Regarding the inclusion of vegetation in an environment, it generates psychological restoration, as has been stressed by Kaplan and Kaplan (1989) and Ulrich et al. (1991). The quantity and diversity of the vegetation are essential characteristics related to a higher environmental preference (Lorenzo et al., 2016). According to Markevych et al. (2017), green spaces improve human health through three functions: reducing harm (e.g., diminishing the impact of the weather on people), restoring capacities (e.g., recovery from stress), and building capacities (e.g., allowing physical and social activities). The possibility of designing built environments that accomplish these three functions needs more research.

Biophilic design is also worthy of mention here since it provides a set of guidelines for creating healthy environments that connect humans with nature (Kellert, 2008). To be considered biophilic, a building should possess qualities such as coherence, complexity, visual contact with nature, and biomorphic patterns (Berto and Barbiero, 2017). Besides the visual experiences of nature, the aural, olfactive, and tactile experiences of nature have also proved beneficial for human beings (Franco et al., 2017).

In other respects, the extent of space visible from a place has also been studied and connected to wellbeing. Places with higher visibility and openness are more restorative and preferred (Herzog et al., 2003; Martínez-Soto et al., 2021). Furthermore, a higher openness in both natural and urban settings relates to lower perceived danger (Herzog and Chernick, 2000).

To sum up the key points from the introduction before the subsequent sections and facilitate the comprehension of the study,

1. Emotions manifest in feelings, appraisals, expressive behavior, actions, language, and physiology, and the pleasure-displeasure dimension is the most characteristic aspect of emotions.
2. Sensory experiences are how stimuli are felt through the senses and body. Unfortunately, contemporary built environments lack sensory richness.
3. Socialization, the physical transformation of a place, and the exploration of it are common types of human activities realized in an environment. The sense of agency allows human beings to be aware of their actions and that they are performing them.
4. Consciousness is the awareness of our internal and external worlds, allowing the existence of subjective experiences. Such experiences convey what it is like to have them; first-person reports are the only way to study human experiences.
5. Research on experiences of places is crucial for understanding how environments can be designed to provide wellbeing.

### 1.6 Objectives

The present exploratory and descriptive study has the following objectives:

1. To present a data collection technique for the inquiry of subjective experiences of places that have been little studied in real environments, such as those related to the emotional, sensory, bodily, interactive, and social aspects of human encounters with places.

2. To obtain a deep understanding of the qualities of a real place and the experiences that such a place evokes in people through the presented technique.

3. To discover how the different experiences afforded by the selected place correlate to each other and to place preference, and generate a map of the experiences of the place based on their correlations.

## 2 Methods

### 2.1 Participants and case selection

Fifty-four participants visited the place (Obispado building esplanade), and 45 completed the surveys correctly (27 women and 18 men, ages 17–25, mean 18.9). Despite being asked to describe their experiences in detail, several participants gave short written descriptions (some of them used one to three words). Therefore, the data obtained from those participants were excluded from the study. Nineteen participants were architecture students (second semester, from the Autonomous University of Nuevo León-UANL), and the other 26 participants were from 14 different careers or occupations. The architecture students who were willing to participate could invite one or two persons not related to the architecture profession to be part of this research. All of them agreed to participate voluntarily and signed an informed consent before the realization of the study. Participants were told they would be asked to visit a public place and answer a survey for which no previous knowledge was needed. Participants were not given any information about the history or current function of the Obispado building. The visit to the place and the surveys occurred from 9:00 a.m. to around 11:00 a.m. over four days (three mostly sunny days and one cloudy day with drizzle). The temperature varied from 22°C to 29°C.

**Figure 1:**
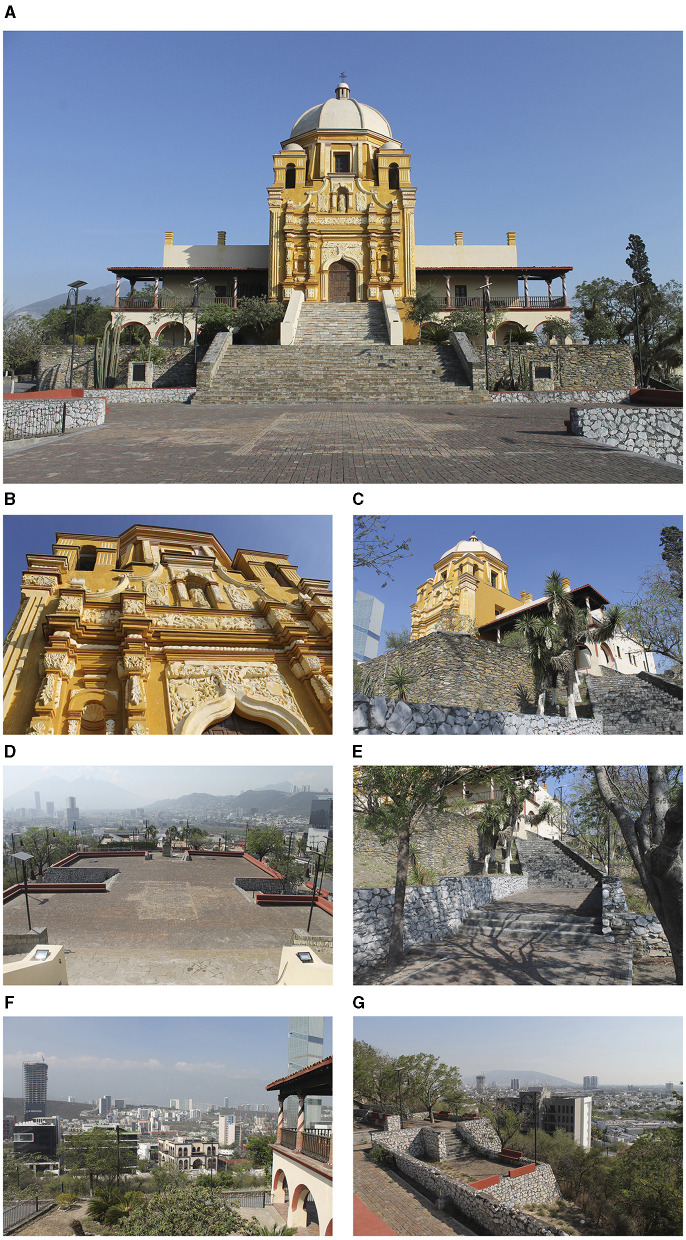
**(A)** Frontal view of the Regional Museum of Nuevo Leon Obispado, Monterrey, Mexico; **(B)** detail of the frontispiece of the oratory; **(C)** Obispado as seen from the east at a lower point; **(D)** the esplanade and the view to the southeast of the cityscape of Monterrey; **(E)** one of the many stairs surrounding the esplanade; **(F)** the galleries of Obispado and the view to the west; and **(G)** view to the north of the city.

**Figure 2:**
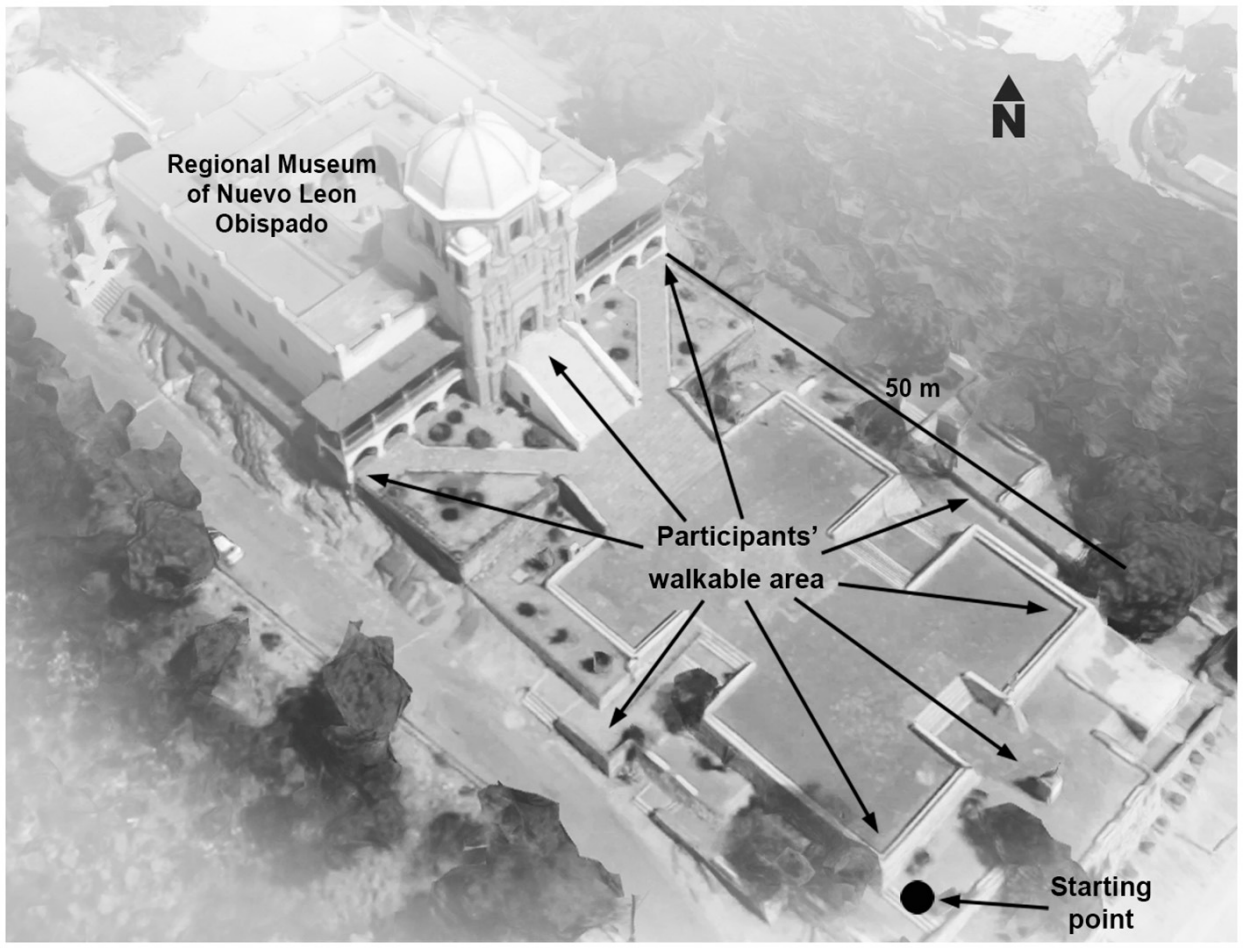
Aerial view of the Obispado building and esplanade. The image was created based on the 3D model of Google Maps.

A historic building located on a hill in Monterrey, Mexico, was selected for this study (Figures 1, 2). The Palace of Our Lady of Guadalupe, as it was initially named, was built for the bishop fray Rafael José Verger from 1787 to 1788 (Flores Salazar, 2001). The building is colloquially named “Obispado,” which means bishopric, due to its original use as the rest house of the Bishop. Currently, Obispado is the Regional Museum of Nuevo Leon. The southeast façade of Obispado (the one visible from the esplanade) has a central body composed of the baroque frontispiece of the oratory and the dome and two superimposed galleries in the laterals (Figure 1). In opposition to the frontispiece, the dome was built during the XIX century, and the galleries were finished in 1999 (Mancillas Hinojosa, 2009). The historical value of the building, the views of the cityscape, the vegetation, and the variety of materials, colors, and ages of the architectural elements made Obispado and its esplanade a suitable case to study a wide range of experiences.

### 2.2 Reactions and Actions Description Survey

RADES is an enabling technique to help the participants describe a broad set of sensory, emotional, and interactive experiences that may occur in a real environment. RADES comprises 120 images of human activities, facial expressions, and bodily positions. Following the terminology of Gombrich (1996), the images of RADES present people's reactions (expressions of inner states) and actions (intentional movements).

**Figure 3:**
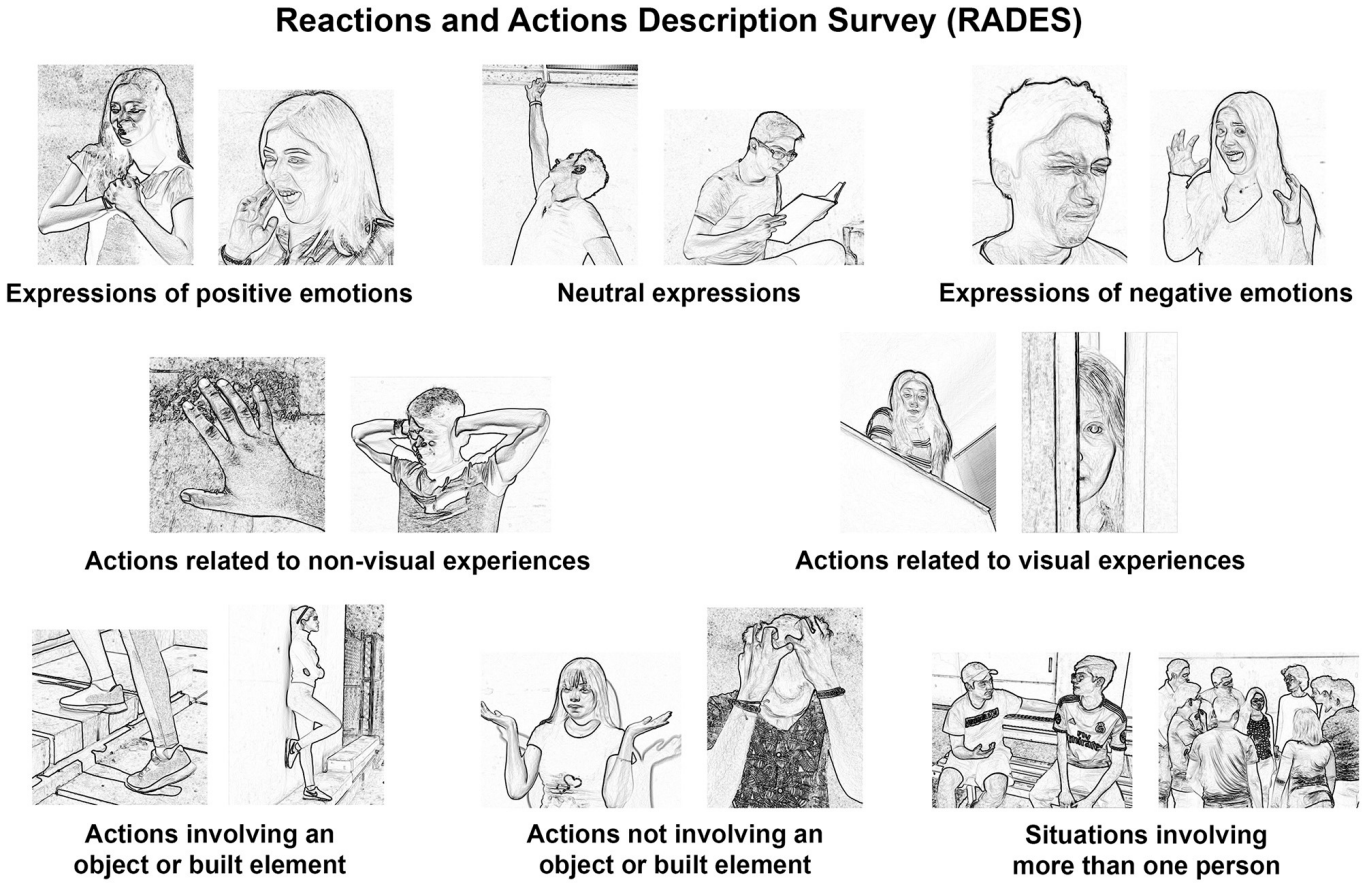
Examples of the images included in the Reactions and Actions Description Survey (RADES), separated by categories.

The images of RADES (Figure 3) were created based on the photographs received from more than one hundred persons who voluntarily participated in the survey creation. The photographs were transformed with edge detection in photo editing software. Edge detection extracts the borders between zones of an image presenting brightness changes, i.e., contrast (Dawson-Howe, 2014). Through this procedure, the photos were transformed to a degree in which the identity of the participants was not discernable. In this regard, the RADES images are similar to sketches since, according to Fish and Scrivener (1990, p. 124), sketches are incomplete structures that “provide ambiguous or indeterminate signs that can provoke innate, unconscious recognition mechanisms to generate a stream of imagery.” Furthermore, since human actions and expressions convey movement, a still image lacks information about what happened before and what will happen next. As Gombrich (1996, p. 121) indicates, “The contorted face of a wrestler may look in isolation as if he were laughing, while a man opening his mouth to eat may appear to be yawning.”

Another relevant characteristic of RADES images is the size in which they are printed—they are ~1 to 1.5 cm per side. The set of images occupies the two sides of a letter-size sheet. The minuscule size of the images, in conjunction with their sketch-like appearance and the lack of movement information, open up the possibilities of their interpretation due to their ambiguity. Since the RADES images are not fixed to one meaning, the participants can comment about an unlimited amount of experiences through the images they choose.

Even though the images are equivocal, RADES was intended to be balanced regarding the types of photos used to create the images (Table 1, Figure 3). Half of the original photos were neutral regarding emotional expressions. The other half of the photos presented expressions associated with emotions (25% depicted expressions of positive emotions and 25%, negative ones). Approximately two-thirds of the photos depicted situations in which a sense distinct to vision was employed, and approximately one-ninth of the photos presented actions predominantly related to visual experiences. Actions or positions involving an object or built element (e.g., to rest on a wall) corresponded to almost half of the photos. Meanwhile, photos of actions and bodily positions not involving an object or built element represented approximately the other half of the total (e.g., touching the forehead as if having a headache). Lastly, approximately one-sixth of the photos presented more than one person, which could be interpreted as socialization or copresence in the place. It is important to note that RADES is not intended to study behavior from an outsider's point of view but to study people's experiences of their actions, movements, and expressions as they occurred in an environment. The variety of images in the survey allows the participants to comment on multiple aspects of their experiences of a place, a broader range of aspects than those they would comment on if they were just asked to talk about their experience without the survey. Pilot tests were carried out in a dozen distinct places before the case study presented in this article to revise and improve the images and instructions of RADES (from 2015 to 2022).

**Table 1:** Categories of photos that originated the images of RADES.

	**Quantity**	**Percentage**
**Categories of expression**		
Neutral expressions.	60	50.0%
Expressions of positive emotions.	30	25.0%
Expressions of negative emotions.	30	25.0%
**Categories of actions related to the use of a sense**		
Actions related to non-visual experiences.	76	63.3%
Actions related to visual experiences.	13	10.8%
Actions not related to the use of a specific sense.	31	25.8%
**Categories of actions related to objects or built elements**		
Actions involving an object or built element.	58	48.3%
Actions not involving an object or built element.	62	51.7%
**Categories about the number of people**		
Situations involving only one person.	99	82.5%
Situations involving more than one person.	21	17.5%

Before applying RADES, participants are asked to walk around and observe the place freely, taking the time they consider necessary. After they finish their tour, they are given the surveys. RADES asks the participants the following: circle an image if during your tour through the place: (a) you felt what the person in the image feels; (b) you did what it is done in the image, or you would have liked to do that; (c) something similar happened to you, or you found yourself in a similar situation to that shown in the image; or (d) something that has to do with the image came to your mind. Furthermore, RADES includes an empty space where the participants can draw an experience they did not find in the survey.

Participants should select at least ten images of RADES to continue with the next part of the survey. The right side of each RADES image presents a space for writing comments and three little rectangular spaces where participants are prompted to put the scores of their experiences. Once the participants have selected all the images related to their experience of the place, they are asked to write a description of the experience on the side of each chosen image. After that activity, they are prompted to ask themselves the following questions:

1. What things I commented on did I notice first, just when I arrived at the place? The participants are asked to put a letter “F” in the comments of experiences that they noted first.

2. What things did I notice when I was already answering the survey? The participants are prompted to write a letter “S” in the comments corresponding to experiences noticed while answering the survey.

3. Which comments are important to me? The participants wrote a letter “I” to all comments they considered important.

4. Which of all the comments are for me the MOST IMPORTANT? The participants were asked to fill in the rectangles at the side of those comments that were the most important of all.

5. Participants are then asked to transform their comments into questions using the expression: How much…? and the statement they wrote, e.g., How much did I feel my foot while walking? Or how much did the place make me feel pensive? Finally, participants answered the questions on a scale from 1 to 10, where 1 indicated “very little,” and 10 indicated “a lot.”

The images of RADES can be used to comment on many types of phenomena. Those comments are later transformed into questions that the participants themselves answer. Therefore, RADES is a flexible technique that transforms according to the participants' experiences.

### 2.3 Environmental Description Survey

The ENVIDES is another enabling technique that allows participants to describe their experiences with a place. While RADES is focused on the person experiencing the place, e.g., his or her emotions or sensations, the purpose of ENVIDES is to help describe the physical setting, its qualities, and meanings. In the Environmental Description Survey (ENVIDES), the participants are asked to complete phrases about the place based on incomplete expressions or stems (de la Fuente Suárez, 2022). Each of the 13 distinct stems appears twice in the survey. The participants complete as many sentences as they have experiences of the place to describe, and several stems may be left blank. Considering that the participants would answer two surveys (RADES and ENVIDES), the ENVIDES created for the present study included only seven stems to reduce the duration of the activity. Six stems were taken from the original version of ENVIDES, and one extra stem, “this view/the views…” was added (Table 2). The stems were selected to allow the participants to comment on the highest quantity of types of elements of the environment with the least quantity of items.

**Table 2:** Stems of the Environmental Description Survey and the eight Positive Experience Statements applied in Obispado.

Environmental Description Survey (ENVIDES)	Saliency, attention, and interest	It stands out…
		I attentively observed… I stared at…
	Liking, pleasure, and preference	I like…
	Place in general and fixed-feature elements	This place…
		This building… These buildings…
		This view… The views…
	Semifixed-feature elements	This object… These things…
Positive Experience Statements (PES)	I really like being in this place.	
	I enjoy exploring this place.	
	It feels very comfortable to be in this place.	
	I like the appearance of this place.	
	I would like to spend my free time in this place.	
	I really like the architecture of this place.	
	I would like to socialize with other people in	
	this place.	
	I enjoy contemplating this place.	

Once the participants completed the stems, they were asked to give additional quantitative data about their experience, similar to what they did during the RADES. They were asked about the experiences they noticed first and those they noticed while answering the survey. They were also asked about the comments they consider important and the most important of all. Then, the participants were asked to transform their comments into questions through the expression: how much…? e.g., How much do I like…? How much did I attentively observe…? Or how much does the building…? Finally, the participants answered the mentally created questions on a scale from 1 to 10.

### 2.4 Positive Experience Statements

A set of Positive Experience Statements (PES) was presented in de la Fuente Suárez (2022). The objective of PES is to obtain assessments of different aspects of place preference (Table 2). After finishing the RADES and ENVIDES, the participants answered the PES section. They were prompted to use a number from 0 to 10 to express how much they agreed with each statement based on their experience of the place, where 10 indicated “completely agree,” and 0 was “completely disagree.”

### 2.5 Quantitative dimensions of the experiences in the RADES and ENVIDES

Both RADES and ENVIDES tasked participants to describe their experiences with words and to rate the quantitative dimensions of immediacy, importance, and intensity of each experience. The order in time in which the participants noticed the experiences is approximated through the dimension of immediacy. What participants noticed or experienced first had a high value of 3 in immediacy. What they experienced while answering the survey had a value of 1. Experiences that did not occur first nor during the survey (empty boxes) assumed an intermediate immediacy value of 2. The personal relevance of each of the experiences that the participants commented on was obtained through the dimension of importance. Experiences considered important obtained a value of 2, while the most important experiences received a value of 3. The comments not considered important (empty boxes) represent a value of 1. Meanwhile, the intensity corresponds to how strong the experiences were for the participants, e.g., how liked, uncomfortable, or relaxed they were. Participants used a 1 to 10 scale to indicate the intensity of each of the experiences they described. The last quantitative dimension of experience is “occurrence,” corresponding to the percentage of participants who commented on a specific experience. Occurrence is the “degree in which a phenomenon is manifested or experienced by people in a place; or capability of the environment to present the experience to people.” (de la Fuente Suárez, 2022, p. 11).

### 2.6 Data analysis

The comments obtained from RADES and ENVIDES were coded, disregarding the specific survey the comments came from. The categorization procedure consisted of the following steps: 1. Creation of two sets of initial categories for the comments obtained, one each for the comments from ENVIDES and RADES. 2. Merger of similar initial categories found through ENVIDES and RADES to create a single set of draft categories based on both techniques (189 draft categories were obtained). 3. Reduction of the number of draft categories to capture the widest variety of experiences with the least number of categories possible. Experience categories with few participants commenting on them (four or fewer) were discarded. A further round of elimination was conducted to remove categories that were too similar (they shared ninety percent of the comments). In this manner, 133 final experience categories were obtained from the comments from RADES and ENVIDES.

Since the experiences commented on by the participants were scored regarding their immediacy, importance, and intensity, the experience categories allowed for the quantitative analysis of the data. After coding the comments into experience categories, the mean values of immediacy, importance, and intensity were calculated for each category (based on the scores of participants who commented on a category).

A Mann-Whitney U test allowed discovering if a significant difference existed between the intensities of the experiences of the architecture students and those of the other participants. The Spearman correlations between the intensities of the experiences were also calculated. The Mann-Whitney U test and the Spearman correlations were realized with the intensity scores of the experiences, in which, if a participant did not comment on an experience category, the intensity of the experience for that participant was scored as zero. The correlations were transformed to distances with the package “psych” in R (Revelle, 2023), and such distances were used to create a multidimensional scaling analysis (MDS) with the package “smacof” in R (de Leeuw and Mair, 2009). In this manner, the MDS presented how the experience categories were located closer or further from each other in space based on their Spearman correlations. The quantitative data of the intensities of each of the experiences deviated significantly from a normal distribution according to the Shapiro-Wilk test realized with the package “stats” in R (R Core Team, 2023). Therefore, the nonparametric tests Mann-Whitney U and Spearman rho were realized with the same package.

## 3 Results

### 3.1 Experience categories and Mann-Whitney U test

The comments obtained from RADES and ENVIDES were organized into 133 experience categories (Tables 3–9). Seventy-five experience categories were entirely or mostly obtained from comments given in the RADES, 57 categories were entirely or mostly obtained from the ENVIDES, and 1 category was obtained from the comments of both surveys in equal parts.

**Table 3:** Strings and experience categories of group 1.-Experiences of the place in general.

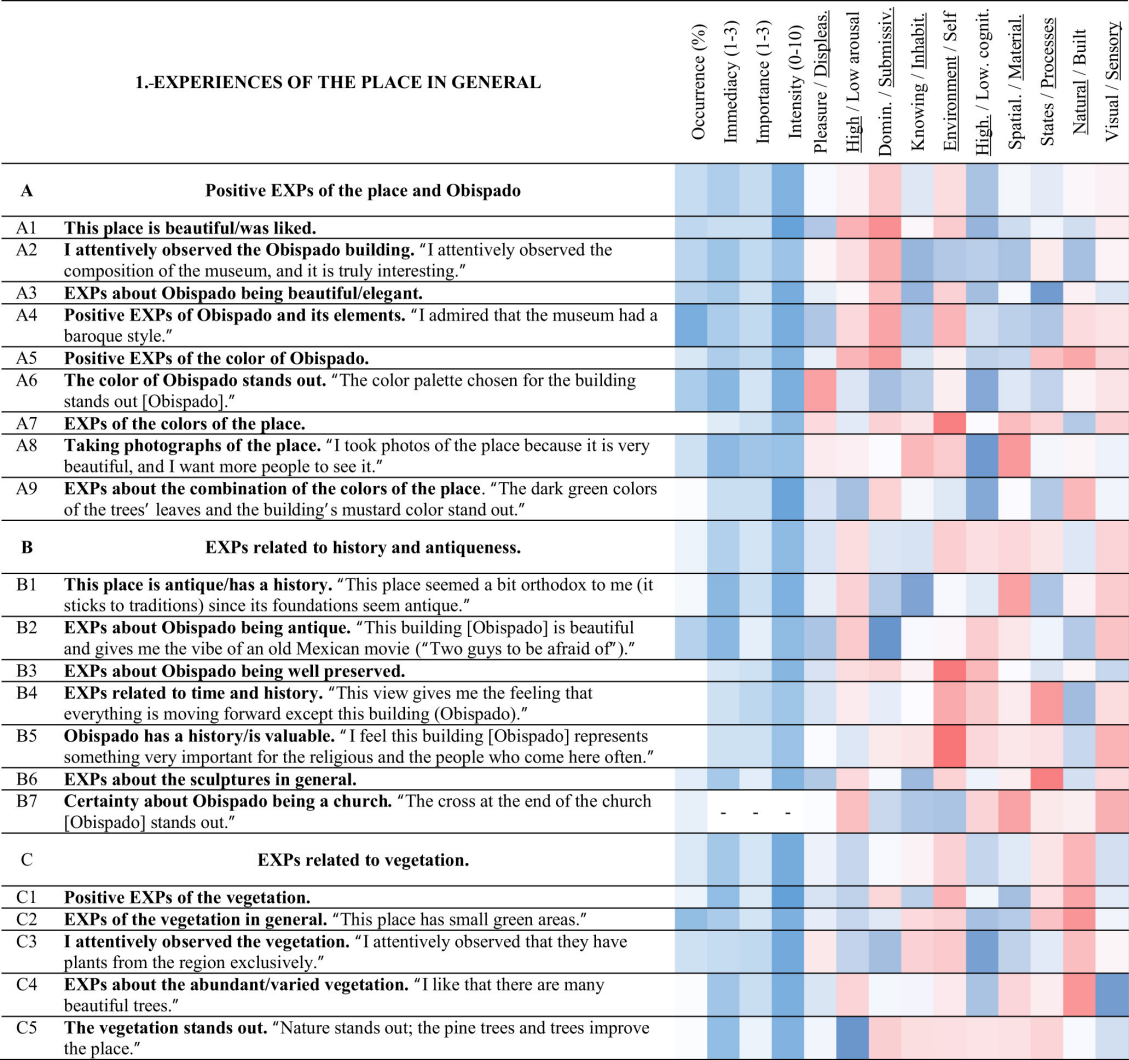

The comments in this table and Tables 4–9 were obtained in Spanish, and some translations are included as examples of the experience categories. The word “experiences” is abbreviated as EXPs in the categories names. Furthermore, the color codes (in this table and Tables 4–9) present the percentage of occurrence and the mean values of immediacy, importance, and intensity for each experience category. The tables also include the values of the 10 dimensions of experience resulting from the MDS (the underlined dimensions appear with red colors). The data presented for the strings are the averages of the values of the experience categories constituting the strings.

Certainty about Obispado being a church < B7> is an experience category indirectly obtained and quantified from the comments. While describing the Obispado, some participants referred to it as a church (certainty=2), and others commented that Obispado looked like a church or doubted whether Obispado was a church (certainty=1). In contrast, others did not express any relation between Obispado and a church (certainty = 0). These three levels of certainty were used to quantify < B7> and to find the correlations with the other experience categories. Since the certainty about Obispado being a church < B7> was not directly commented on by the participants, there were no values for the comments' intensity, immediacy, or importance.

**Table 4:** Strings and experience categories of group 2.-Experiences related to exploration.

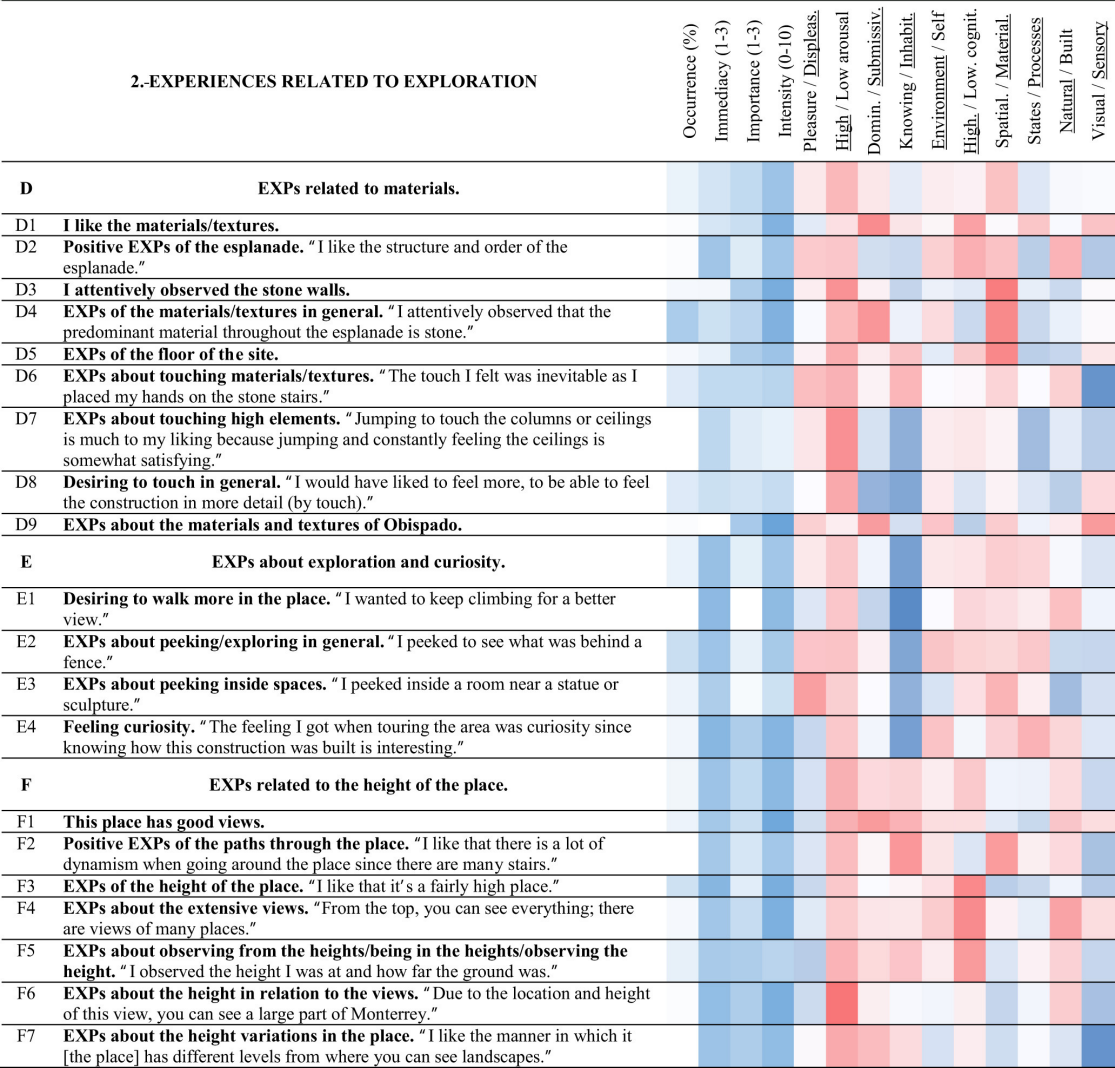

**Table 5:** Strings and experience categories of group 3.-Experiences of the views.

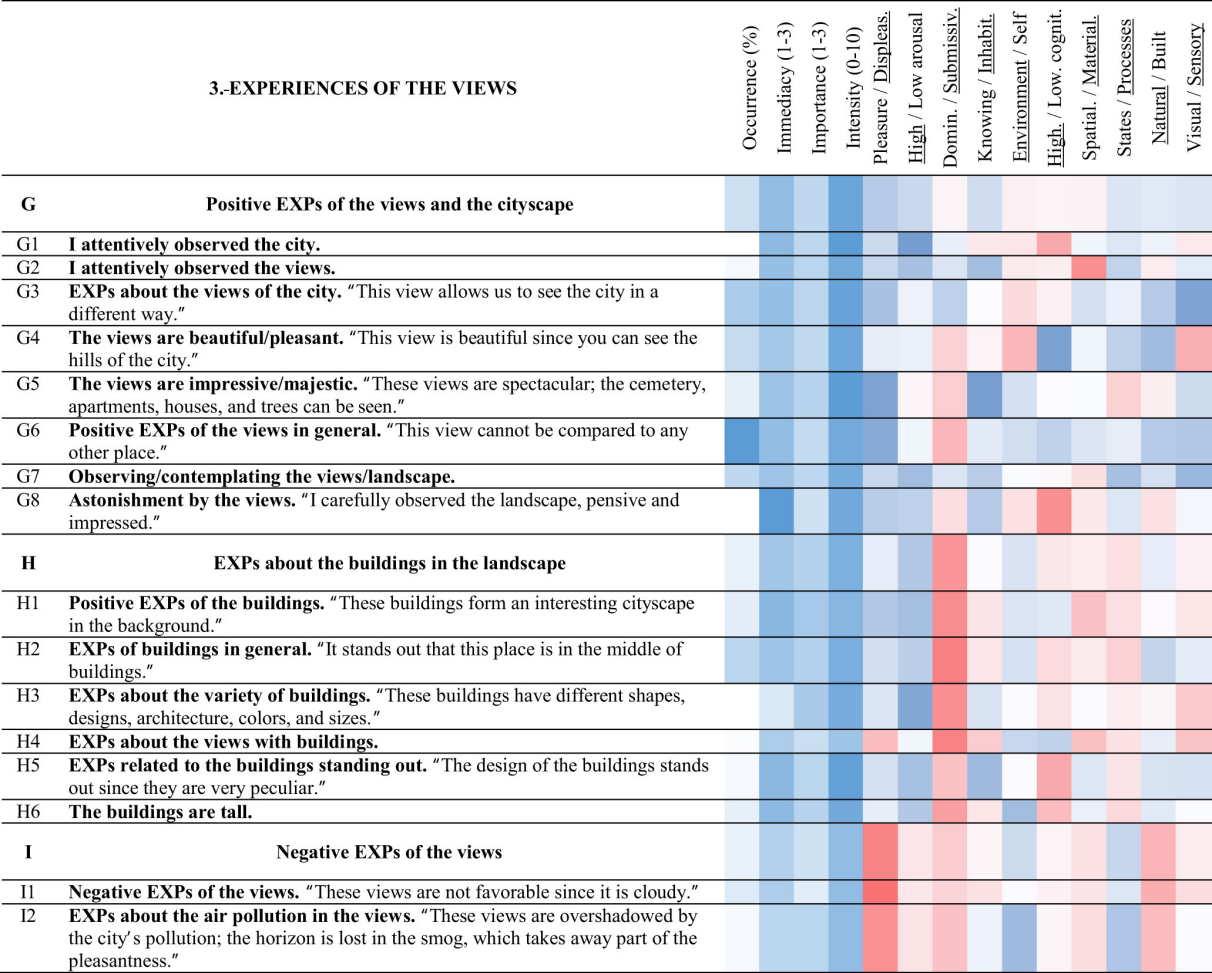

**Table 6:** Strings and experience categories of group 4.-Positive emotional and sensory experiences.

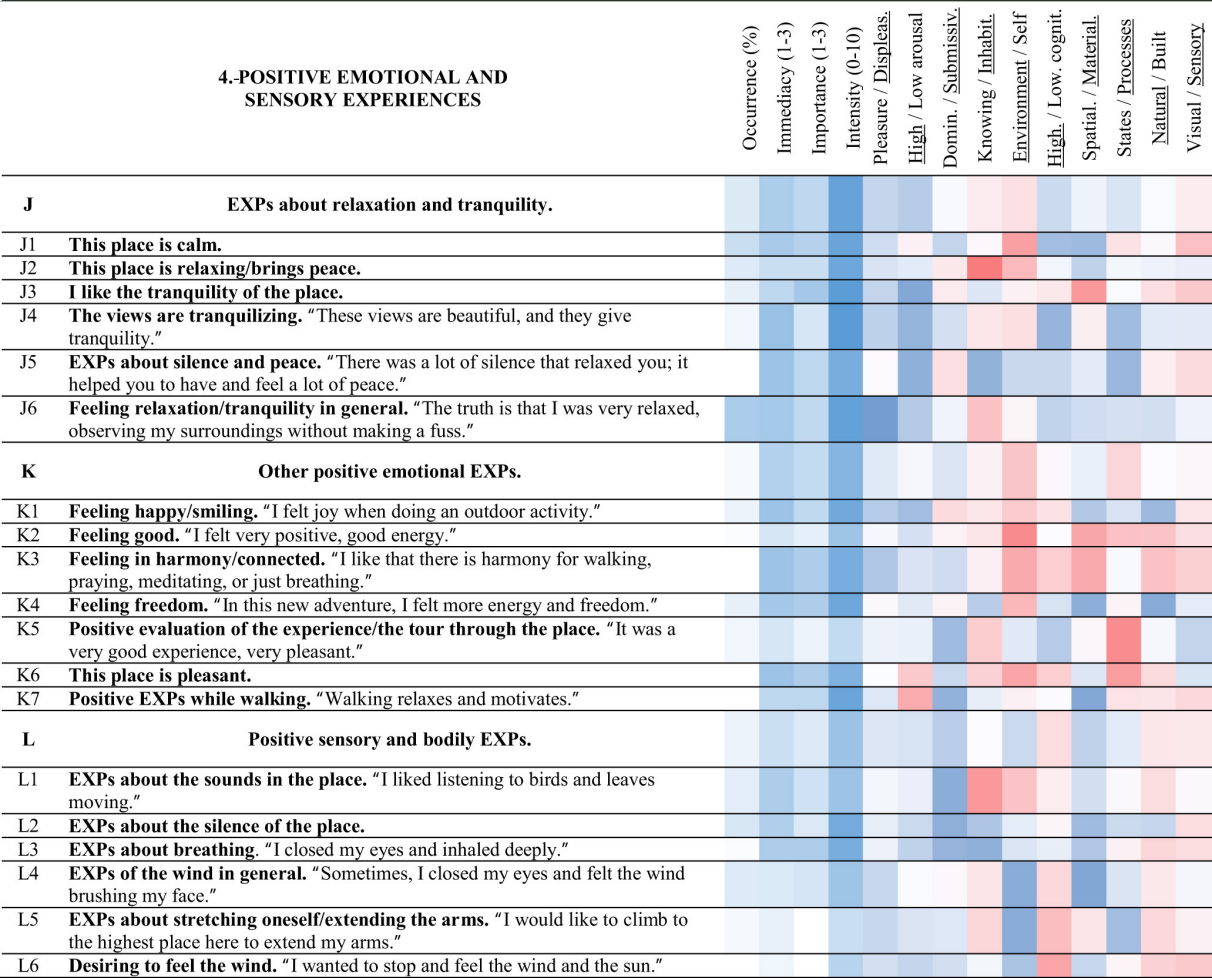

**Table 7:** Strings and experience categories of group 5.-Experiences about social and individual activities.

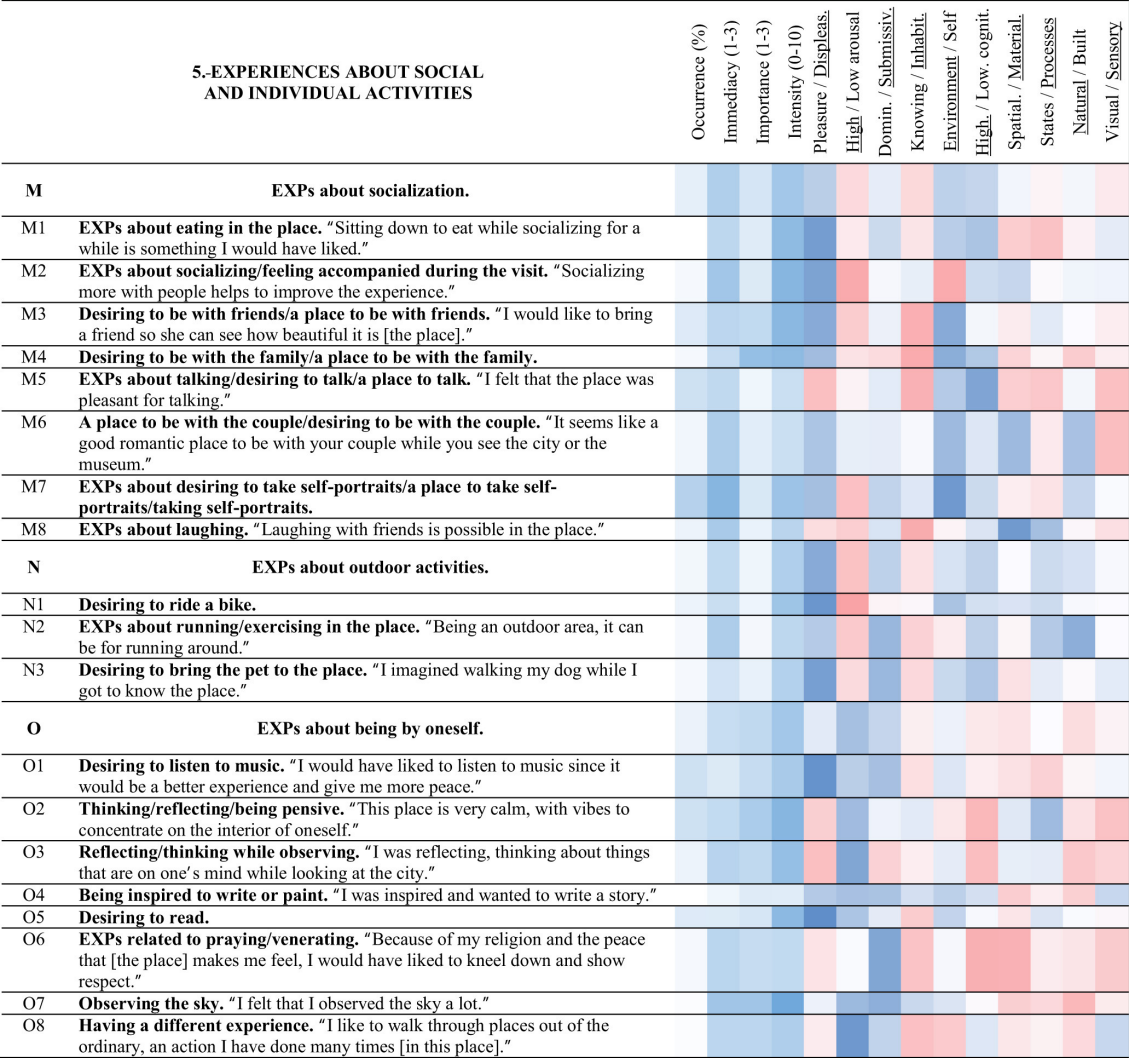

**Table 8:** Strings and experience categories of group 6.-Experiences about observation and space.

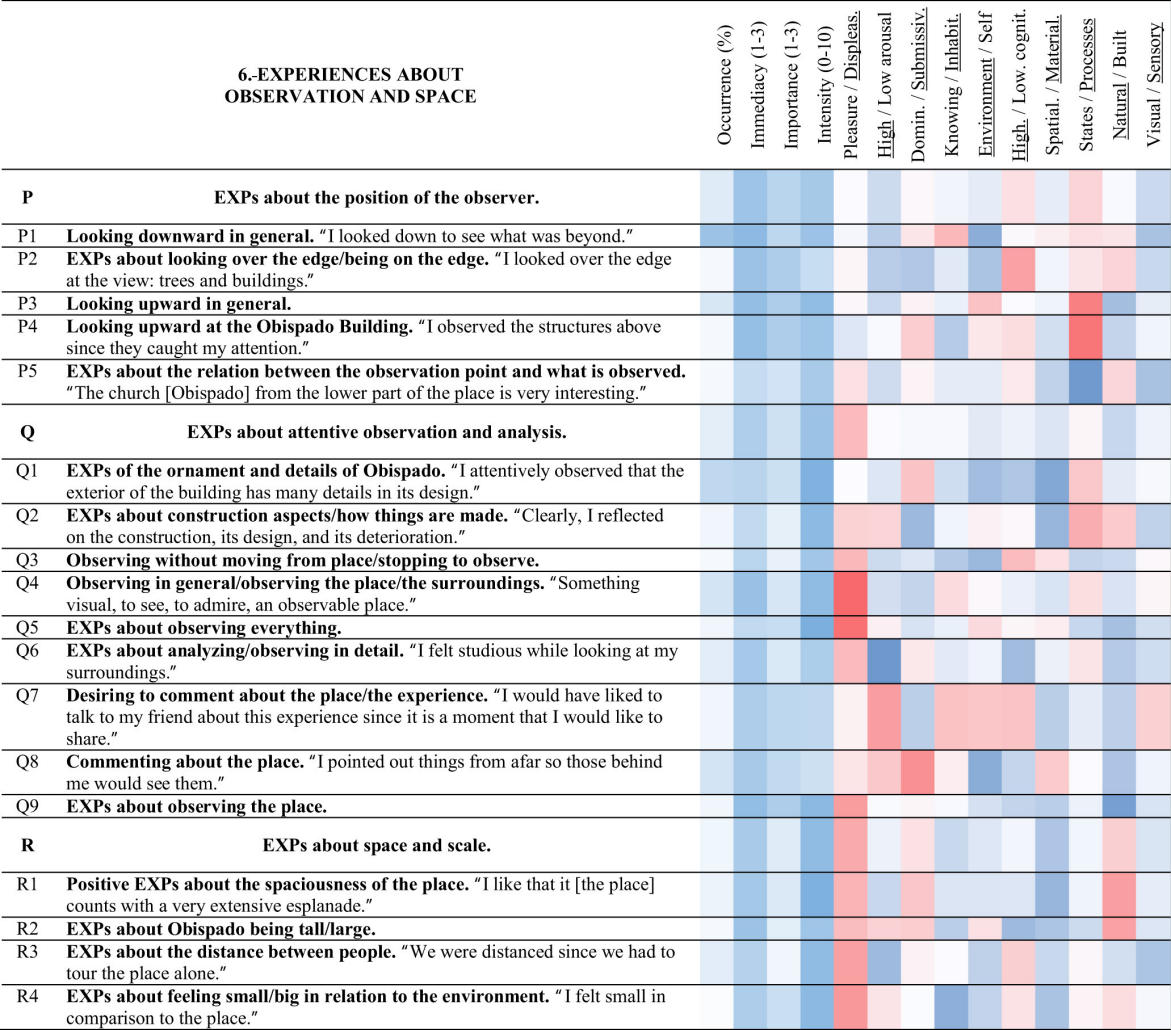

**Table 9:** Strings and experience categories of group 7.-Experiences about effort, discomfort and recovery.

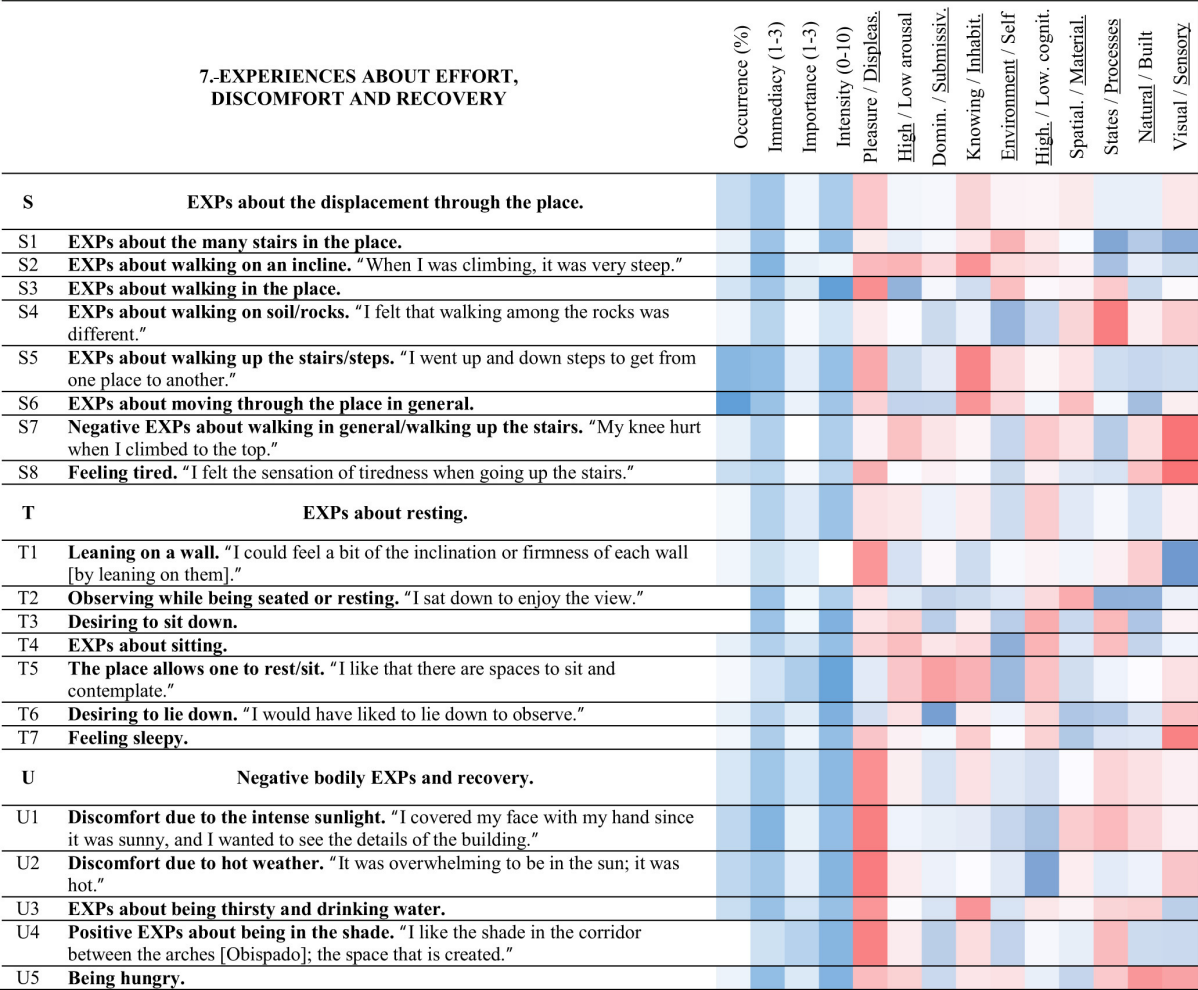

Regarding the intensities of the experience categories, it was found through the Mann-Whitney U test (significance level of 0.05) that only 8 experience categories out of 133 presented a significant difference between the group of architecture students and the group of people from other disciplines and occupations (laypeople). Interestingly, the laypeople significantly experienced the Obispado building as older (EXPs about Obispado being Antique < B2>), perhaps due to their lower exposure to architectural history. Meanwhile, the architecture students scored higher in < Q8> commenting about the place, as if they were more prone to discuss it due to their knowledge of architecture. Furthermore, the students focused more on the materials of the place and the sense of touch, having higher intensities in < D4> EXPs of the materials/textures in general and < D6> EXPs about touching materials/textures.

Even though there was no significant difference between groups regarding how tired they felt, the students had higher intensity experiences of < T4> EXPs about sitting, and they were more aware of their displacement: < S6> EXPs about moving through the place in general, while the laypeople experienced < E1> desiring to walk more in the place. Another difference between groups was found in < J5> EXPs about silence and peace, which the laypeople experienced at a higher level. Regarding PES, none of the eight items differed between groups. Since the experience of the place did not vary considerably between groups, the following sections present the data of all participants without distinguishing between them.

### 3.2 Multidimensional scaling analysis in two dimensions

**Figure 4:**
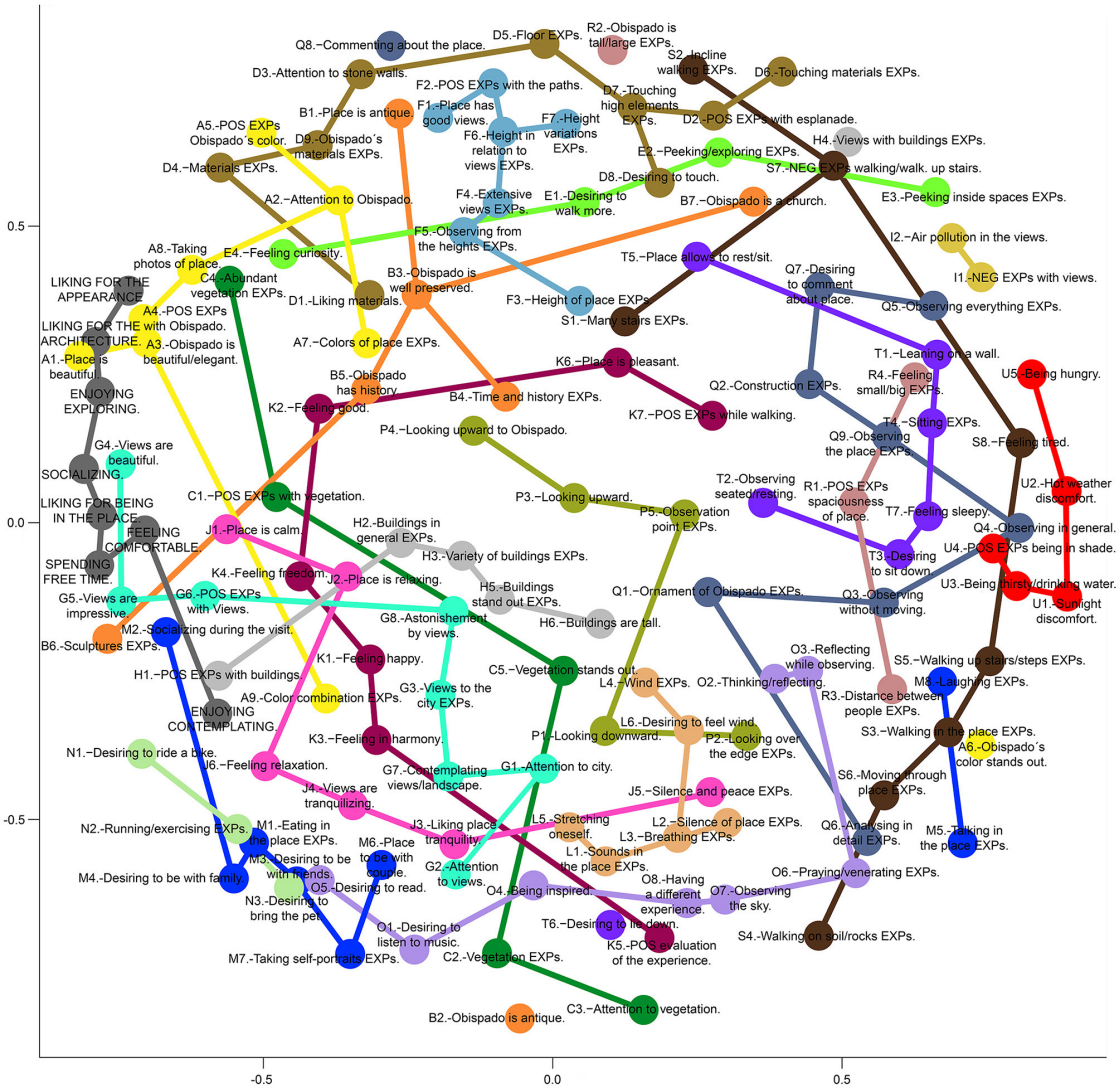
Multidimensional scaling (MDS) of the experience categories presented during the visit to Obispado. The categories are shown with circles or nodes belonging to one of the strings (constellations of nodes distinguishable by their color). The nodes of a string are connected with lines solely to facilitate their visualization.

A bidimensional MDS was created based on the Spearman correlations between 141 dimensions comprising the evaluations of the 8 PES and the intensity values of the 133 experience categories discovered through the surveys (Figure 4). The experience categories were aggregated in 21 Clusters, which are called strings, due to their elongated configuration. The proximity of the nodes of the experience categories in the MDS (a quantitative aspect) and the similarity between the categories (a qualitative aspect) were considered to create the strings. Furthermore, seven groups were composed by including three strings in each of them. Tables 3–9 present all the groups with the corresponding strings and experience categories reported in the place.

The x-axis of the MDS presents a clear opposition between experiences positively related to place preference on the left and experiences negatively related to place preference on the right. Experiences that appear in the center of the horizontal axis present a neutral relation with place preference. The upper part of the diagram presents experiences focused on the place, its material qualities, and architectural elements; they are the “experiences of the place.” Exploration, interest in the history of the place, aesthetic experiences of the place or Obispado, or the enjoyment of the materials and colors are examples of experiences of the upper part. Meanwhile, the lower part of the diagram illustrates experiences that, while afforded by the environment, do not focus on the place itself or its qualities. They are the “other experiences occurring in the place” that include a wide range of experiences, from the contemplation of the landscape that is visible from the place (but beyond its limits) to the experiences focused on the activities that could be realized on the site.

### 3.3 Statistics of the experience categories

**Figure 5:**
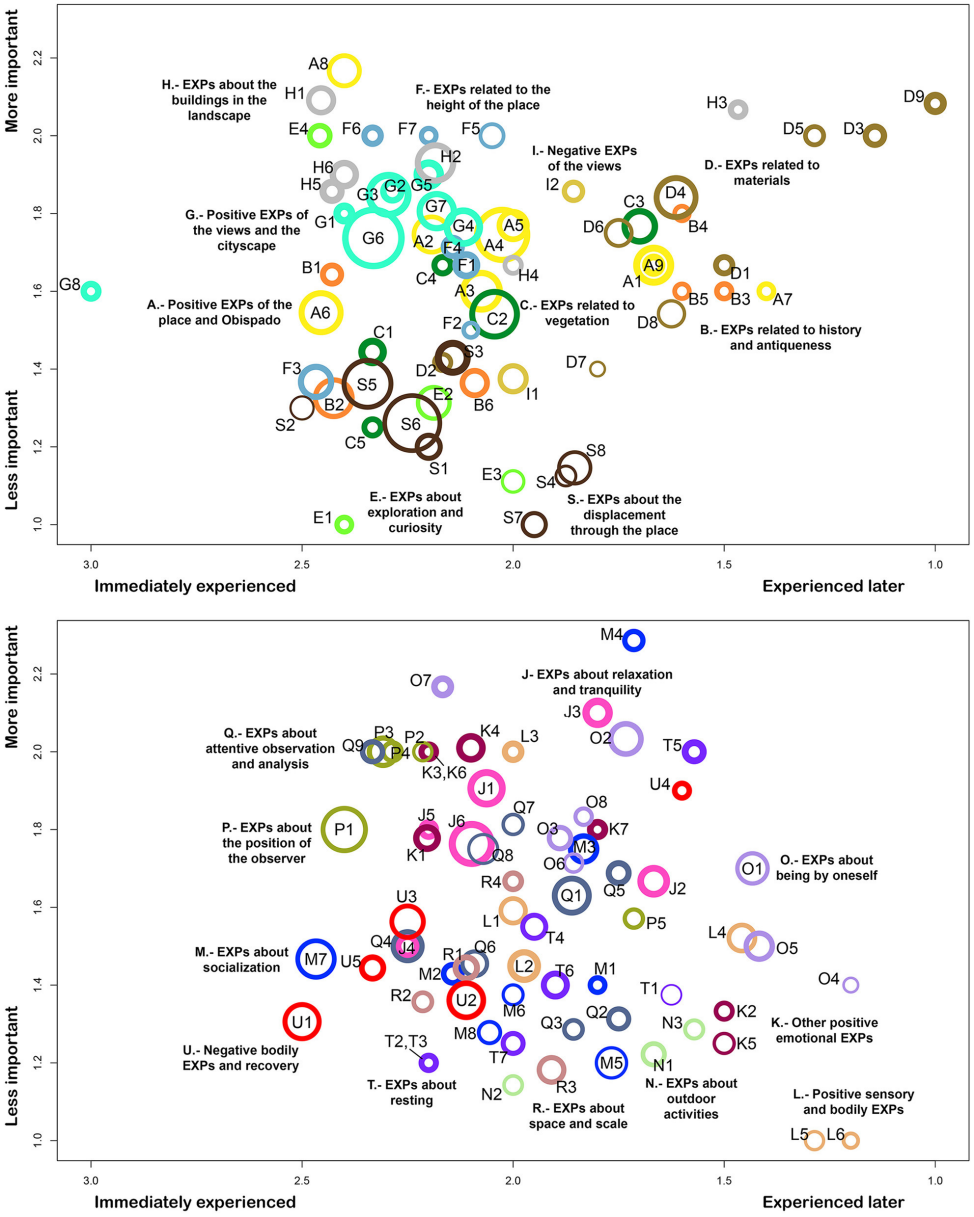
Experience Scatterplots (Immediacy/Importance) of the Opispado. The dimension of immediacy is the x-axis, which ranges from the more immediately experienced on the left to the less immediately experienced on the right. The importance is shown through the y-axis. The occurrence of the experiences corresponds to the size of the circles. Meanwhile, the intensity is presented with the circles' thickness. The experience categories were divided into two scatterplots to prevent cluttering.

**Figure 6:**
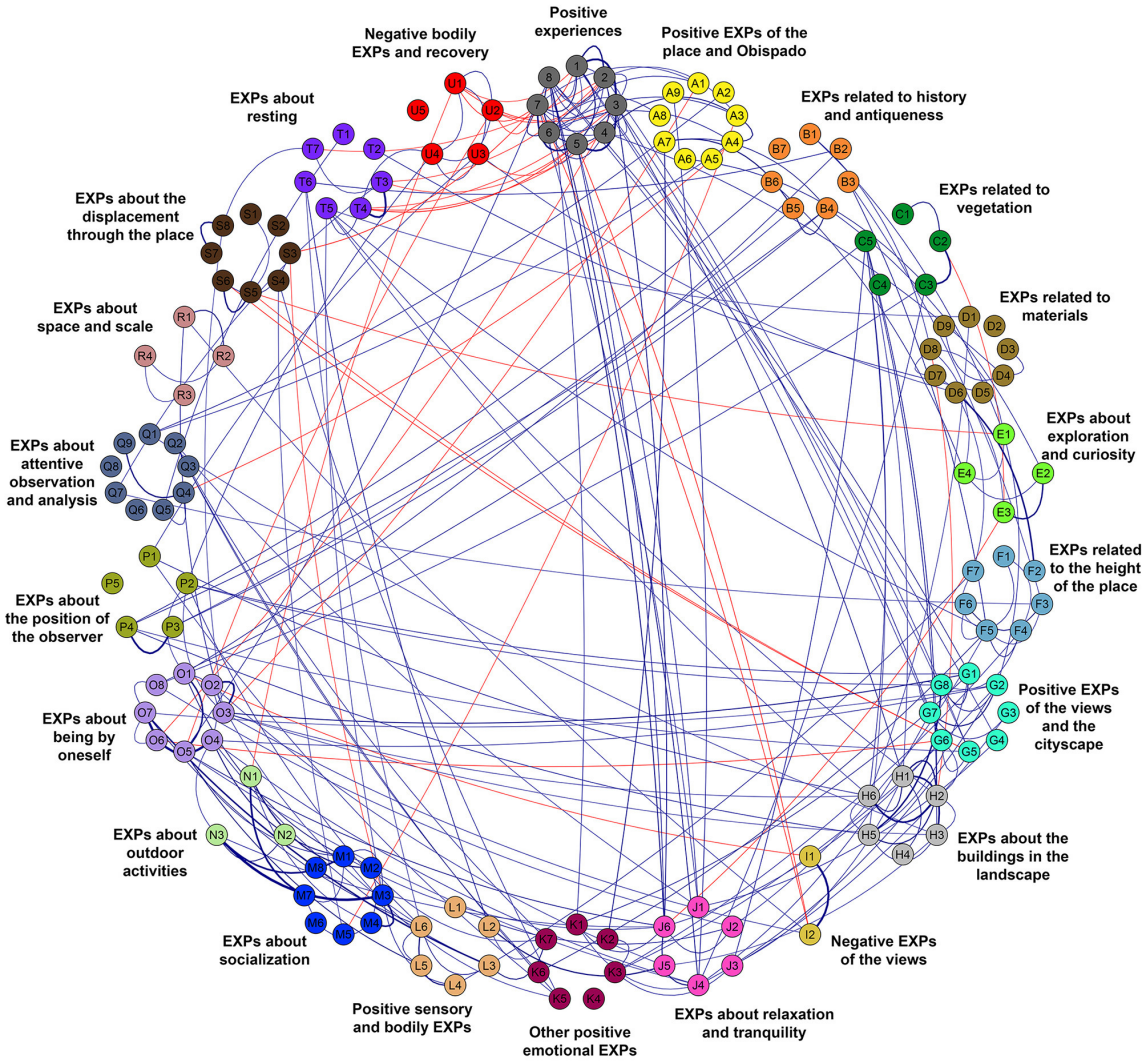
The correlation network showing significant Spearman correlations of 0.38 and higher. The experience categories (nodes) are grouped into strings (circles of nodes). The thickness of the edges represents the strength of the correlation, while the curvature of the edge is higher when the connected nodes are closer.

The occurrence and the mean values of immediacy, importance, and intensity of the experiences can be found in Tables 3–9 and are graphically represented in Figure 5. Meanwhile, Figure 6 presents the Spearman correlations between experiences. The following sections present some of the most insightful descriptive statistics and correlations (significant at 0.05 level).

#### 3.3.1 Experiences of the place in general (1)

##### 3.3.1.1 Positive EXPs of the place and Obispado (A)

String A (yellow in Figures 4–6) includes the positive experiences had with the place and Obispado, with some of these experiences focused on the colors. Positive EXPs of Obispado and its elements < A4> was the most commented experience of this group (71%), followed by the color of Obispado stands out < A6> (49%). This place is beautiful/was liked < A1> was related to < A4> positive EXPs of Obispado and its elements (0.35). EXPs of the colors of the place < A7> was related to Obispado has a history/is valuable < B5> (0.55) and < B4> EXPs related to time and history (0.50). I attentively observed the Obispado building < A2> related to < Q1> EXPs of the ornament and details of Obispado (0.46).

##### 3.3.1.2 EXPs related to history and antiqueness (B)

The experiences about the age and history of the place and the Obispado building were tagged under string B (orange). EXPs about Obispado being antique < B2> was the most commented experience of this category (44%). This experience < B2> had negative correlations with < A5> positive EXPs of the color of Obispado (−0.33), and < A3> EXPs about Obispado being beautiful/elegant (−0.30). This place is antique/has a history < B1>, and < B7> certainty about Obispado being a church, were related to < E1> desiring to walk more in the place (0.44 and 0.33). EXPs about the sculptures in general < B6> was related to < E4> feeling curiosity (0.48). EXPs about desiring to take self-portraits/a place to take self-portraits/taking self-portraits < M7> was related to < B2> EXPs about Obispado being antique (0.36).

##### 3.3.1.3 EXPs related to vegetation (C)

All the comments related to the vegetation in the visited site or present in the landscape were categorized under string C (dark green). Sixty percent of the participants commented on EXPs of the vegetation in general < C2>. Positive EXPs of the vegetation < C1> was an intense experience (9 of 10). The vegetation stands out < C5> was correlated to < H5> EXPs related to the buildings standing out (0.40).

#### 3.3.2 Experiences related to exploration (2)

##### 3.3.2.1 EXPs related to materials (D)

String D (brown) includes experiences, whether visual or tactile, related to the materials of the Obispado building, the stone walls, or the floor of the esplanade. Approximately half of the participants commented about < D4> EXPs of the materials/textures in general (49%). Approximately one-fourth of the participants reported on < D6> EXPs about touching materials/textures (27%) and < D8> desiring to touch in general (27%). String D includes the last experiences noticed during the visit: < D9> EXPs about the materials and textures of Obispado, and < D3> I attentively observed the stone walls (immediacy of 1 and 1.1). The latter experience < D3> was related to < D8> desiring to touch in general (0.32). Not only the tactile experiences were related to the presence of textured materials, but also to the antiqueness of the place. Desiring to touch in general < D8> was related to < B1> this place is antique/has a history (0.42), < B2> EXPs about Obispado being antique (0.30), and < E1> desiring to walk more in the place (0.40).

##### 3.3.2.2 EXPs about exploration and curiosity (E)

The experiences related to the inspection of the place and the curiosity felt were included in string E (green). Such experiences were noticed early during the visit. The most commented experience of this category was < E2> EXPs about peeking/exploring in general (36%).

Feeling curiosity < E4> was related to < D8> desiring to touch in general (0.40), < C4> EXPs about the abundant/varied vegetation (0.31), and < A5> positive EXPs of the color of Obispado (0.30). EXPs about peeking/exploring in general < E2> was related to < D8> desiring to touch in general (0.31), and < B4> EXPs related to time and history (0.43). EXPs about peeking/exploring in general < E2> was opposed to < O5> desiring to read (−0.35) and < M4> desiring to be with the family/a place to be with the family (−0.31). The higher < E3> EXPs about peeking inside spaces, the lower < J6> feeling relaxation/tranquility in general (−0.41), < A4> positive EXPs of Obispado and its elements (−0.32), and < C2> EXPs of the vegetation in general (−0.40).

##### 3.3.2.3 EXPs related to the height of the place (F)

String F (pale blue) includes experiences about the site's height, the variety of heights found during the itinerary, and the extensive views encountered. When noticed, the experiences of string F tended to be experienced early. The experience with the highest occurrence of this category was < F3> EXPs of the height of the place (33%). The higher the intensity of < F4> EXPs about the extensive views, the more < G8> astonishment by the views (0.42), and < L5> EXPs about stretching oneself/extending the arms (0.31). Positive EXPs of the paths through the place < F2> related to < D6> EXPs about touching materials/textures (0.62).

#### 3.3.3 Experiences of the views (3)

##### 3.3.3.1 Positive EXPs of the views and the cityscape (G)

String G (cyan) refers to the experiences related to the views and the positive experiences that arose from their observation. With 84% of occurrence, positive EXPs of the views in general < G6> was the most commented experience of all. Meanwhile, astonishment by the views < G8> was the most immediate experience generated on this site (3 out of 3). Accordingly, string G had the highest mean immediacy of all the strings (2.4 out of 3). I attentively observed the city < G1> presented a high intensity of 9.2 out of 10. In general, the experiences of string G tended to be immediate, important, and intense.

Astonishment by the views < G8> related to < G1> I attentively observed the city (0.52), < P2> EXPs about looking over the edge/being on the edge (0.42), and < O3> reflecting/thinking while observing (0.36). The latter experience < O3> was also related to < G1> I attentively observed the city (0.53). In addition, the higher the intensity of < G1> I attentively observed the city, the higher < K1> feeling happy/smiling (0.46).

##### 3.3.3.2 EXPs about the buildings in the landscape (H)

String H (light gray) corresponds to the experiences related to the buildings visible from the site (mainly towers). EXPs of buildings in general < H2> was the most commented experience in this category (42%). Meanwhile, EXPs related to the buildings standing out < H5> was an intense experience (9 out of 10). The buildings in the landscape played an important role in < G8> astonishment by the views, since it correlated to < H5> EXPs related to the buildings standing out (0.61), < H6> the buildings are tall (0.35), and < H3> EXPs about the variety of buildings (0.33).

##### 3.3.3.3 Negative EXPs of the views (I)

The negative aspects of the views compose the string I (mustard). The experiences included in this string were less intense than those in string G (positive EXPs of the views and the cityscape). Regarding the correlations: the more intense < I2> EXPs about the air pollution in the views, the more < O3> reflecting/thinking while observing (0.40). Negative EXPs of the views < I1> negatively related to < O1> desiring to listen to music (−0.41), < T6> desiring to lie down (−0.31), and < O5> desiring to read (−0.35).

#### 3.3.4 Positive emotional and sensory experiences (4)

##### 3.3.4.1 EXPs about relaxation and tranquility (J)

String J (magenta) is about the relaxation felt during the visit and the tranquility of the place. Feeling relaxation/tranquility in general < J6> was the experience with the highest occurrence of this string (49%). The views are tranquilizing < J4> was the most intense experience had in the place (9.3 of 10), and string J was the string with the highest mean intensity of all.

Feeling relaxation/tranquility in general < J6> related to < G6> positive EXPs of the views in general (0.42). Feeling relaxation/tranquility in general < J6> also related to < O5> desiring to read, < M1> EXPs about eating in the place, and < O1> desiring to listen to music (0.50, 0.41, and 0.35). Feeling relaxation/tranquility in general < J6> and feeling happy/smiling < K1> were also correlated (0.48). Feeling relaxation/tranquility in general < J6> was inversely related to < S7> negative EXPs about walking in general/walking up the stairs (−0.33). Meanwhile, I like the tranquility of the place < J3> correlated with < K3> feeling in harmony/connected (0.43).

##### 3.3.4.2 Other positive emotional EXPs (K)

Positive emotions generated in the place unrelated to relaxation are included in string K (dark magenta). Compared with those of string J.-EXPs about relaxation and tranquility, experiences of string K were commented on less. Feeling freedom < K4> had the highest occurrence of this string (22%) and was related to < C1> positive EXPs of the vegetation (0.31).

Feeling in harmony/connected < K3> related to < G2> I attentively observed the views (0.42), and < F4> EXPs about the extensive views (0.40). Feeling good < K2> related to < A7> EXPs of the colors of the place (0.52), but the more < A6> the color of Obispado stands out, the less < K3> feeling in harmony/connected (−0.32). Meanwhile, the more < C5> the vegetation stands out, the more < K3> feeling in harmony/connected (0.30).

##### 3.3.4.3 Positive sensory and bodily EXPs (L)

String L (salmon) encompasses the experiences noticed in the place that involved senses different from sight and were evaluated positively. EXPs about the silence of the place < L2> was the experience with the highest occurrence of string L (29%). EXPs about the silence of the place < L2> related to < L4> EXPs of the wind in general (0.34), and < K7> positive EXPs while walking (0.42). Meanwhile, the higher < L1> EXPs about the sounds in the place, the higher < J2> this place is relaxing/brings peace (0.48), and < K3> feeling in harmony/connected (0.33).

EXPs about breathing < L3> related to < O7> observing the sky (0.61), < L6> desiring to feel the wind (0.50), < K7> positive EXPs while walking (0.53), and < O4> being inspired to write or paint (0.46). EXPs about stretching oneself/extending the arms < L5> was related to < O5> desiring to read (0.53) and < L6> desiring to feel the wind (0.48). EXPs of the wind in general < L4> related to < F5> EXPs about observing from the heights/being in the heights/observing the height (0.34). Similarly, EXPs about looking over the edge/being on the edge < P2> related to < L6> desiring to feel the wind (0.44).

#### 3.3.5 Experiences about social and individual activities (5)

##### 3.3.5.1 EXPs about socialization (M)

String M (blue) includes the experiences of the interaction with other people that took place during the visit to the site, or that could occur in it. Desiring to be with the family/a place to be with the family < M4> was the most important experience presented during the visit to the place (2.3 of 3). Meanwhile, EXPs about desiring to take self-portraits/a place to take self-portraits/taking self-portraits < M7> was the experience with the highest occurrence of this string (44%). Desiring to be with friends/a place to be with friends < M3> was related to < T5> the place allows one to rest/sit (0.40). A place to be with the couple/desiring to be with the couple < M6> was related to < K4> feeling freedom (0.36) and < K1> feeling happy/smiling (0.36). Additionally, the more < M2> EXPs about socializing/feeling accompanied during the visit, the less < S8> feeling tired (−0.31).

##### 3.3.5.2 EXPs about outdoor activities (N)

Leisure and exercise activities compose string N (light green). The experiences of this string were of low importance and intensity. Desiring to bring the pet to the place < N3> was strongly correlated to < M3> desiring to be with friends/a place to be with friends (0.76). Desiring to bring the pet to the place < N3> was also related to < N2> EXPs about running/exercising in the place (0.52), and < J6> feeling relaxation/tranquility in general (0.42). EXPs about running/exercising in the place < N2> related to < S6> EXPs about moving through the place in general, and < S1> EXPs about the many stairs in the place (0.35 and 0.32 respectively).

##### 3.3.5.3 EXPs about being by oneself (O)

String O (light violet) includes experiences and activities commonly occurring while alone. Thinking/reflecting/being pensive < O2> and desiring to listen to music < O1> were the experiences with the highest occurrence in this string (both presented a 33%). Regarding the correlations, being inspired to write or paint < O4> was strongly correlated with < O7> observing the sky (0.73), and it also correlated with < G2> I attentively observed the views (0.43). The less < A1> this place is beautiful/was liked, the more < O7> observing the sky, and the more < O3> reflecting/thinking while observing (−0.31, and −0.39). Positive EXPs about the spaciousness of the place < R1> was also related to < O3> reflecting/thinking while observing (0.48). Desiring to listen to music < O1> was related to < B2> EXPs about Obispado being antique (0.46). Thinking/reflecting/being pensive < O2> was related to < J5> EXPs about silence and peace (0.39). Meanwhile, observing the sky < O7> was connected to < G2> I attentively observed the views (0.39), < O6> EXPs related to praying/venerating (0.38), and also to < L6> desiring to feel the wind (0.50).

#### 3.3.6 Experiences about observation and space (6)

##### 3.3.6.1 EXPs about the position of the observer (P)

String P (olive) encompasses the experiences in which the participants noticed their position and location in space while observing. Looking downward in general < P1> was the experience with the highest occurrence of this string (56%). Meanwhile, < P3> looking upward in general was commented on by 29% of the participants. Looking downward in general < P1> related to < K5> positive evaluation of the experience/the tour through the place (0.40), < J6> feeling relaxation/tranquility in general (0.32), and < O4> being inspired to write or paint (0.37). Looking upward at the Obispado building < P4> correlated with < B4> EXPs related to time and history (0.47). EXPs about the relation between the observation point and what is observed < P5> correlated with < Q3> observing without moving from place/stopping to observe (0.30), and < T1> leaning on a wall (0.32).

##### 3.3.6.2 EXPs about attentive observation and analysis (Q)

String Q (blue-gray) is about the act of observation and the exhaustive analysis of the place and its elements. EXPs of the ornament and details of Obispado < Q1> presented the highest occurrence of the experiences of string Q (40%). Desiring to comment about the place/the experience < Q7> related to < F3> EXPs of the height of the place (0.38), < B2> EXPs about Obispado being antique (0.36), and < B4> EXPs related to time and history (0.32). Observing in general/observing the place/the surroundings < Q4> was related to < O2> thinking/reflecting/being pensive (0.31), and < Q2> EXPs about construction aspects/how things are made (0.34). EXPs about analyzing/observing in detail < Q6> was positively related to < Q1> EXPs of the ornament and details of Obispado (0.32). Lastly, EXPs about observing everything < Q5> related to < E3> EXPs about peeking inside spaces (0.36).

##### 3.3.6.3 EXPs about space and scale (R)

String R (pink) is a heterogeneous group that includes experiences that connect space, distance, scale, and people. EXPs about the distance between people < R3> was the most commented experience of this string (24%). EXPs about feeling small/big in relation to the environment < R4> correlated with < R2> EXPs about Obispado being tall/large (0.40), and < Q3> observing without moving from place/stopping to observe (0.38). In addition, positive EXPs about the spaciousness of the place < R1> correlated with < R3> EXPs about the distance between people (0.32).

#### 3.3.7 Experiences about effort, discomfort and recovery (7)

##### 3.3.7.1 EXPs about the displacement through the place (S)

The experiences about walking through the place and the effort and tiredness that such an activity conveyed compose the string S (dark Brown). Two experiences of this category presented very high occurrence; 82% of the participants reported < S6> EXPs about moving through the place in general, and 64% reported < S5> EXPs about walking up the stairs/steps. Nevertheless, the experiences of string S tended to be of very little importance and intensity. EXPs about walking up the stairs/steps < S5> related negatively to < E1> desiring to walk more in the place (−0.41) and < E4> feeling curiosity (−0.36). Contrarily, EXPs about walking on soil/rocks < S4> were related to < K5> positive evaluation of the experience/the tour through the place (0.43); while < S1> EXPs about the many stairs in the place, related to < O8> having a different experience (0.32).

##### 3.3.7.2 EXPs about resting (T)

The experiences related to sitting, resting, or feeling sleepy compose the string T (violet). Most of the experiences of this string were of little occurrence. Observing while being seated or resting < T2> was related to < D5> EXPs of the floor of the site (0.44). Desiring to sit down < T3> and observing without moving from the place/stopping to observe < Q3> were related to < T7> feeling sleepy (0.40 and 0.30). Feeling sleepy < T7>, in turn, was related to < S8> feeling tired (0.40). Desiring to lie down < T6> related to < L2> EXPs about the silence of the place (0.49), and < L6> desiring to feel the wind (0.39). Lastly, EXPs about sitting < T4> was connected to < U4> positive EXPs about being in the shade (0.44).

##### 3.3.7.3 Negative bodily EXPs and recovery (U)

String U (red) includes the uncomfortable sensory and bodily experiences the participants had in the place and the experiences of how they found relief from them. Discomfort due to the intense sunlight < U1> and discomfort due to hot weather < U2> presented 40% of occurrence. The more discomfort due to the intense sunlight < U1>, and discomfort due to hot weather < U2>, the more < U4> positive EXPs about being in the shade (both correlations of 0.43). Discomfort due to the intense sunlight < U1>, and EXPs about being thirsty and drinking water < U3> were both negatively related to < A1> this place is beautiful/was liked (−0.34 and −0.37). Similarly, the more discomfort due to hot weather < U2>, the less < O5> desiring to read (−0.39). Positive EXPs about being in the shade < U4> related to < L1> EXPs about the sounds in the place (0.31). Meanwhile, the more < U5> being hungry, the more < M5> EXPs about talking/desiring to talk/a place to talk (0.34), and the less < J6> feeling relaxation/tranquility in general (−0.37).

### 3.4 The Positive Experience Statements and their correlations with the experience categories

Four PES items received a moderately high average evaluation of 8.0 out of 10: enjoying exploring, enjoying contemplating, liking for the appearance, and liking for the architecture. Meanwhile, spending free time received the lowest score of 6.0. The other three items received scores between 7.2 to 7.9. Six PES presented significant correlations with < A3> EXPs about Obispado being beautiful/elegant; liking for the appearance was the one with the highest correlation (0.53). Liking for the architecture was related to < A1> this place is beautiful/was liked (0.47), and < A3> EXPs about Obispado being beautiful/elegant (0.48). Liking for the architecture also presented a correlation with < A8> taking photographs of the place (0.43).

EXPs of the materials/textures in general < D4> had correlations with five PES; liking for the appearance was the one with the highest correlation (0.44). Respecting < C1> positive EXPs of the vegetation, it presented three significant correlations with PES (liking for being in the place was the highest with 0.32). In a similar manner, EXPs about the abundant/varied vegetation < C4> was related to enjoying exploring (0.30). The higher enjoying exploring, the less < S6> EXPs about moving through the place in general (−0.37), < S8> feeling tired (−0.36), or < T7> feeling sleepy (−0.38). Furthermore, the more enjoying exploring, the less < M5> EXPs about talking/desiring to talk/a place to talk (−0.36). A high level of engagement in the exploration of the place may have reduced the other experiences mentioned above. Meanwhile, desiring to sit down < T3> had six negative correlations with the PES items, and observing without moving from place/stopping to observe < Q3> was negatively correlated with three items. Those facts may indicate that a low preference for the place was accompanied by sitting or observing statically.

Observing the sky < O7> was negatively related to liking for the appearance and liking for the architecture (−0.35 and −0.32). Seemingly, the participants having an aesthetic experience of the place focused less on the sky during their visit. The more liking for the appearance, the less < Q4> observing in general/observing the place/the surroundings (−0.33). Just as during a higher enjoyment of exploration, the displacement through space is less experienced, during a higher liking of the appearance of the place, the experience of observing is less noticed. In other respects, a correlation was present between feeling comfortable and < A9> EXPs about the combination of the colors of the place (0.32).

Regarding the views, < G9> positive EXPs of the views in general positively impacted place preference since five PES presented correlations with such experience. Meanwhile, the views are impressive/majestic < G5> had a positive correlation with enjoying exploring (0.40). Regarding enjoying contemplating, it was related to < G1> I attentively observed the city (0.49), and < G4> the views are beautiful/pleasant (0.41). The views are tranquilizing < J4> was related to three PES; remarkably, it related to enjoying contemplating (0.52). Furthermore, enjoying contemplating related to < K1> feeling happy/smiling (0.45), < O5> desiring to read (0.45), and < M6> a place to be with the couple/desiring to be with the couple (0.30), and was inversely related to < E1> desiring to walk more in the place (−0.35). Contrarily, EXPs about the air pollution in the views < J2> could have had a negative impact on the preference for the place since spending free time, socializing, and enjoying contemplating had negative correlations with such experience (−0.45 −0.43, and −0.35).

This place is calm < J1> was positively related to three PES, from which spending free time and socializing stand out (0.45 and 0.43). Five PES presented correlations with < J6> feeling relaxation/tranquility in general. The relations of < J6> with feeling comfortable, enjoying contemplating, and spending free time were especially remarkable (0.52, 0.53, and 0.48). Meanwhile, discomfort due to intense sunlight < U1> had negative relations with feeling comfortable (−0.44), and socializing (−0.46). The less feeling comfortable, the more < U4> positive EXPs about being in the shade (−0.33). The positive experience < U4> may be strongly experienced after the discomfort of being in the place without a shade. As Erwine (2017, p. 118) indicates, “The only way to experience pleasure is to move from a state of discomfort to a state of comfort.” Therefore, a positive experience such as < U4> is related to the negative experiences of the weather and appears on the right side of the MDS (Figure 4), with the other experiences negatively related to the PES. Regarding < U2> discomfort due to hot weather, it had a negative impact on the preference for the place since it presented six negative correlations with the PES items.

Regarding socializing, it was inversely related to the physical activity realized during the visit: < S3> EXPs about walking in the place (−0.47), < S5> EXPs about walking up the stairs/steps (−0.32), and < S6> EXPs about moving through the place in general (−0.32). Meanwhile, EXPs about socializing/feeling accompanied during the visit < M2> related to spending free time (0.33), and socializing (0.31). Meanwhile, positive EXPs about the spaciousness of the place < R1> was negatively related to socializing (−0.37). Table 10 summarizes the correlations between the strings of experience and the PES.

**Table 10:** Quantity of positive and negative significant correlations between the experience categories and the Positive Experience Statements (per string).

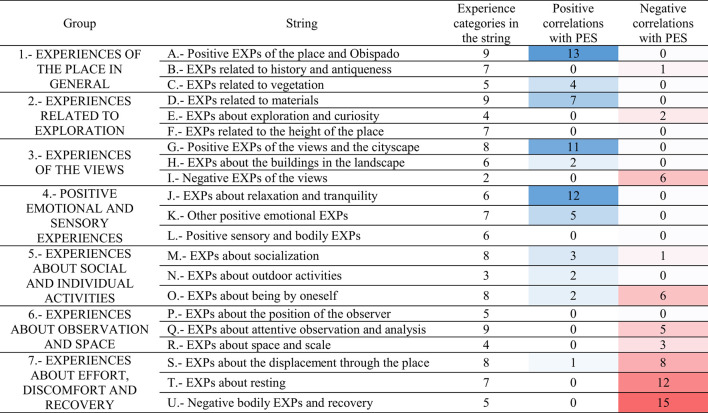

### 3.5 Multidimensional scaling analysis in 10 dimensions

Several MDS with three or more dimensions were created to explore the inherent dimensions of the experience of the visited place. Those MDS only included the 133 experience categories based on the participants' comments without the items from the PES. The ten-dimensional MDS alone is described here since it presents the highest number of dimensions that are interpretable. The data of the 10 dimensions per experience category are shown in Tables 3–9.

The first axis of the MDS is pleasure/displeasure, which implies the degree to which the experiences of the place involve a positive or negative effect. G.-Positive EXPs of the views and the cityscape, M.-EXPs about socialization, and N.-EXPs about outdoor activities are primarily located on the PLEASURE side of the axis. Meanwhile, I.-Negative EXPs of the views, Q.-EXPs about attentive observation and analysis, and U.-Negative bodily EXPs and recovery represent the displeasure side.

The second axis corresponds to high arousal/low arousal, which separates the experiences involving more mental or physical activation from the experiences of lower activation. Experiences of high arousal are found in D.-EXPs related to materials, E.-EXPs about exploration and curiosity, and F.-EXPs related to the height of the place. In other respects, low arousal experiences may be found in J.-EXPs about relaxation and tranquility and O.-EXPs about being by oneself. Several experiences of environmental elements are located on the low arousal side of the axis. These experiences, while not about calmed states, presumably generate such states: C.-EXPs related to vegetation, and G.-Positive EXPs of the views and the cityscape. The relationship between vegetation and relaxation has been studied in de la Fuente Suárez and Martínez-Soto (2022).

Dominance/submissiveness is the third axis, and it presents the opposition between the experiences involving control and autonomy of the person and the experiences related to aspects in which the person has no control. Some of the experiences in L.-Positive sensory and bodily EXPs, N.-EXPs about outdoor activities, and O.-EXPs about being by oneself represent the dominance side of the axis. Contrarily, all the experiences in H.-EXPs about the buildings in the landscape, and I.-Negative EXPs of the views, and several experiences in A.-Positive EXPs of the place and Obispado, and R.-EXPs about space and scale, represent the submissiveness side of the axis. These SUBMISSIVENESS strings relate to environmental aspects of high potency (EPA), such as the large scale of Obispado and the cityscape buildings. It is noticeable that the three first axes of the MDS are the dimensions of the PAD model (Russell and Mehrabian, 1977) and are related to the dimensions of the EPA model (Osgood et al., 1957).

The fourth axis is knowing-oriented/inhabiting-oriented, which puts to one side the experiences related to the place as an object of knowledge and, to the other, the experiences of livability and the possibilities for activities and socialization. The knowing-oriented side of the axis presents all the experiences in E.-EXPs about exploration and curiosity, and some other experiences as those in A.-Positive EXPs of the place and Obispado, and G.-Positive EXPs of the views and the cityscape. Meanwhile, the inhabiting-oriented side is represented by some of the experiences in F.-EXPs related to the height of the place, M.-EXPs about socialization., O.-EXPs about being by oneself, and S.-EXPs about the displacement through the place.

The fifth axis is environment-focused/self-focused, which distinguishes between experiences centered on external objects or the place and those centered on some aspect of the experiencing person. Several experiences of the strings: A.-Positive EXPs of the place and Obispado, B.-EXPs related to history and antiqueness, and K.-Other positive emotional EXPs appear on the environment-focused side of the axis. Meanwhile, the self-focused side includes experiences of the following strings: L.-Positive sensory and bodily EXPs, M.-EXPs about socialization, and T.-EXPs about resting.

Higher cognition/lower cognition is the sixth axis. According to König et al. (2013), reasoning, language, planning, and decision-making are high-level cognitive processes, while sensory processing is a low-level cognitive process. Following this distinction, the experiences involving thoughts, desires, and intentions of action correspond to the higher cognition side of this axis. Some of the last experiences are in B.-EXPs related to history and antiqueness, F.-EXPs related to the height of the place, O.-EXPs about being by oneself, and T.-EXPs about resting. Meanwhile, the experiences related to perceptions and sensations occupy the lower cognition side of the axis. The strings presenting experiences in the lower cognition side are A.-Positive EXPs of the place and Obispado, in which the colors of the place played an important role, C.-EXPs related to vegetation, and U.-Negative bodily EXPs and recovery, in which the hot weather was a relevant aspect.

The seventh axis is spatiality-focused/materiality-focused, which is the axis more related to the elements configuring a place. On the one hand, this axis presents the experiences of the space, its extension, and atmosphere, and on the other, the experiences of the material elements and their qualities. The experiences in R.-EXPs about space and scale, in which the spaciousness of the place is noteworthy, are located on the spatiality-focused side of the axis. On the same side, some experiences related to the atmosphere and the feeling of the space appear in K.-Other positive emotional EXPs, which includes the feeling of freedom, and L.-Positive sensory and bodily EXPs, in which the wind and the breathing were relevant. Meanwhile, D.-EXPs related to materials, is the string representing the materiality-focused side. It is important to pinpoint that the trends that guided the interpretation of spatiality/materiality and the following three axes were not as well defined as those of the other dimensions presented.

The eighth axis, states-focused/processes-focused, distinguishes the experiences of what is fixed and independent of time from the events and transformations that involve time. Some experiences in A.-Positive EXPs of the place and Obispado, and G.-Positive EXPs of the views and the cityscape are on the states-focused side. Meanwhile, the processes-focused side is represented by some experiences in K.-Other positive emotional EXPs, and U.-Negative bodily EXPs and recovery.

The ninth axis of experience is natural-focused/built-focused, which marks the difference between the experiences mainly focused on the vegetation, the sky, or the wind and those having the buildings and other human-made elements as their content. While C.-EXPs related to vegetation represents the natural-focused side, the built-focused side includes several experiences in B.-EXPs related to history and antiqueness, G.-Positive EXPs of the views and the cityscape, and Q.-EXPs about attentive observation and analysis.

Lastly, the tenth axis, visual/sensory, separates the experiences obtained through vision from the experiences involving any other of the senses. The visual side of the axis is represented by F.-EXPs related to the height of the place, G.-Positive EXPs of the views and the cityscape, and P.-EXPs about the position of the observer. Meanwhile, several experiences in B.-EXPs related to history and antiqueness, J.-EXPs about relaxation and tranquility, and S.-EXPs about the displacement through the place can be found on the sensory side.

The categories of emotional, sensory, and interactive experiences were used in the introduction to specify the types of experiences RADES intended to detect. Those categories can be found in several of the MDS dimensions. The first three dimensions of the MDS relate to the emotional aspects of experience, and the sensory aspects are separated from the visual ones in the last of the obtained dimensions. Concurrently, the knowing/inhabiting dimension distinguishes between the two main experiences linked to people's actions in an environment, whether those related to exploration or those about using the place and socializing in it.

In synthesis, five of the dimensions found are about the “how” of a place's experiences, i.e., they are related to the affective and cognitive aspects. These are pleasure, arousal, dominance, knowing/inhabiting, and higher/lower cognition. Meanwhile, the other five dimensions are about the “what” of the experiences, i.e., their content—environment/self, spatiality/materiality, states/processes, natural/built, and visual/sensory.

## 4 Discussion

The following section is divided into two parts. The first discusses the data collection technique introduced in this article (corresponding to objective 1), and the second focuses on the experiences of the place uncovered through this technique (objectives 2 and 3).

### 4.1 The data collection technique

The present study is based on written descriptions of experiences obtained after the participants explored a place. ENVIDES focuses on describing the site, its elements, and its qualities and depends solely on words for giving such comments. Meanwhile, RADES opens up the possibility of selecting images related to a wide range of sensory, emotional, and interactive experiences that people are aware of during the visit to a place. The advantages of using RADES can be summarized in the following points:

1. The obtention of a panorama of the experience of a place. This study explored the contents of consciousness as they emerged in a place and were naturally experienced by people. Most existing qualitative and quantitative techniques for the study of experience focus on specific aspects of it. In opposition, RADES does not ask participants to comment on what they perceive through each sense or what things in the place caused the emotions presented in a list; on the contrary, participants select and answer only the items related to their experience of the place. In this manner, the presented technique allows for studying environmental and architectural experiences without forcing participants to analyze or intellectualize their experiences. RADES helped to discover a multiplicity of experiences beyond the ones related to the material and visual aspects of the visited site. The present study is perhaps the first to simultaneously inquire into a broad range of experiences occurring in a real place. Therefore, this article contributes to the existing knowledge about the experience of a place and the emotions and sensations people feel precisely by studying them in an ecologically valid context.

2. A single technique to collect qualitative and quantitative data. Besides the written descriptions, RADES allows us to obtain ratings about the intensity and importance of the experiences described, which can be analyzed quantitatively. It is important to pinpoint that one limitation of the proposed technique is that the comments obtained are less rich in subtleties of experience description than those obtained through interviews. Nevertheless, ENVIDES and RADES enable the researcher to obtain many comments in a shorter time than interviews.

3. The exploration of connections between the experiences and place preference. The intensities of the distinct experience categories obtained through RADES and ENVIDES may be used to find the aspects of the place that correlate positively or negatively with place preference. Regarding the correlations between experience categories, such as the one between < D8> desiring to touch in general and < E4> feeling curiosity, it could be tempting to hypothesize that such experiences may have accompanied each other. Nevertheless, the fact that a correlation was found does not indicate that these experiences simultaneously occurred since they could have emerged at different stages of the visit to the place. Similarly, it cannot be asserted from a correlation that one experience caused another. Further research should be realized regarding the possible interpretations of the correlations between experiences discovered through RADES and ENVIDES.

4. A versatile technique for studying human-environment relations. RADES can be helpful in comparing the experiences of different groups of people in the same place, e.g., locals and tourists, young people and elders, or experienced architects and laypeople. Besides being a data collection technique, RADES is an easy-to-explain activity that can be enjoyable and be used with children. The images of RADES are focused on the person experiencing the place and are not architectural in themselves. Therefore, this technique can be used in any type of environment, including natural ones in which no buildings are present. Furthermore, since RADES allows participants to describe positive, negative, or neutral experiences, this technique can be used to understand the dynamics of experiences of places that do not cause interest in people or places that generate mainly negative experiences.

The subjective experiences of places and their varieties compose a broad area of study that must be investigated in future research. Therefore, more data collection techniques should be created and contrasted with those employed here.

### 4.2 The experiences of a place

Comments about the beauty and antiqueness of the Obispado building were prominent. The color of Obispado was mentioned positively, and it was experienced as standing out in the place. The participants expressed curiosity and interest in exploring the place. Attention was given to the materials and textures of Obispado and the stone walls, and some tactile experiences occurred. Participants had positive experiences about the views and cityscape, expressing enjoyment and astonishment. The towers and other buildings, together with the vegetation, were notable features contributing to the site's overall experience.

Furthermore, the site facilitated a range of social activities altogether with individual experiences of reflection. Feelings of relaxation and tranquility were prevalent among participants, and positive sensory experiences were noted, such as those related to breathing and the silence of the place. In other respects, participants experienced physical exertion, especially while climbing stairs, but also found opportunities for resting. It is important to note that the results might be influenced by how young adults engage with the environment, and then the results could be specific to such a group of participants.

Based on the correlations between the experience categories, a set of 10 dimensions was discovered which correspond to the main aspects of the phenomenology of the place. These include pleasure/displeasure, high/low arousal, dominance/submissiveness, knowing/inhabiting, environment/self, higher/lower cognition, spatiality/materiality, states/processes, natural/built, and visual/sensory. The MDS indicates that the dimensions of an experience occurring in a human encounter with a place encompass the affective and cognitive aspects in conjunction with the contents of such experience. Presumably, these dimensions show how people's external and internal worlds, from built elements and natural sounds to curiosity and pensiveness, compete for being the contents of consciousness. For example, the more sensory experiences occupy space in consciousness, the less visual experiences do.

The discovered dimensions resulted from data collected through techniques applied to participants immersed in a real environment. Therefore, such dimensions cannot be obtained in studies presenting participants with virtual reality scenes or photographs of places. More studies should be conducted to verify if such a ten-dimensional scheme can also be found in other case studies, or on the contrary, if every place and space possesses a particular configuration of experiences in the MDS. A model of environmental experience could be created if the dimensions obtained here appear in other places with quite distinct characteristics. The model's dimensions of experience could be employed not only to map the experience categories of a single environment but also to map the environments themselves. Some places could generate more pleasurable, sensory, or other types of experiences than others. The environments in specific zones of the model (e.g., the high arousal/environment-focused/visual zone) could generate specific emotional and cognitive states in people, which could be favorable under certain circumstances.

The fact that the axes of the MDS of Obispado are pretty well defined means that the case study offers a wide variety of experiences. Obispado, its esplanade, and the views compose an enriched environment experienced as a little world. Based on the above, two questions can be posed: what kind of benefit can be gained from an environment with such a variety of experience possibilities? Secondly, what are the consequences of places not offering experiential variety for human beings? The ten experience axes' implications for environmental design should be studied further.

In other respects, the experiences of Obispado could be analyzed considering the three aspects that generate a healthy environment presented by Markevych et al. (2017)—restoring capacities, building capacities, and reducing harm. The presence of vegetation, the extensive views of the cityscape, and the experiences related to relaxation commented by the participants indicate that the visit to Obispado was a restorative experience. Furthermore, the esplanade of Obispado, with the possibilities for physical and social interaction that it offers, and the Obispado building generating interest in the history of the place, are qualities building capacities in people. Nevertheless, the hot weather and the intense sunlight are two aspects that reduce place preference and need mitigation (reducing harm).

In this study, the participants were confronted with a real place in which the weather conditions, the blazing sun, the scarcity of shades in the center of the esplanade, and the pollution in the landscape were part of the experience. Therefore, the context of the experience of the place was drastically different from that of an interior space with a controlled atmosphere. This study remarked on how such demanding conditions affected the other experiences of the place, and it is one of its contributions. In the displeasing experiences, there may be possibilities for improvement and intervention to the site, e.g., by introducing shading devices. Nonetheless, it is essential to remark that such intervention would change the experiences of the place and the correlations between them. In this case, the new elements could affect the experiences of the Obispado building and the city views. Further research is needed to know how the results of this type of study should be applied in architecture and urban design for the benefit of people and the preservation of the identity of the place.

More research is needed to determine how the experiences of a place relate to psychological restoration and health. Furthermore, the importance of color in generating positive mood states and the relation of the materials, the desire to touch, and curiosity should be studied further and revalued in contemporary architecture practice. This study was based on the principle that the improvement of inhabitable environments should be based on research encompassing more than the physical qualities of places and considering the totality of aspects of a human being. The data collection techniques, statistical analysis, and visualization proposed here may be helpful for architects and designers who want to become more capable of distinguishing beneficial qualities of the environment to be employed during the design of inhabitable places.

## Data availability statement

The raw data supporting the conclusions of this article will be made available by the authors, without undue reservation.

## Ethics statement

Ethical review and approval was not required for this study in accordance with the local legislation and institutional requirements. The participants provided their written informed consent to participate in this study.

## Author contributions

LF: Conceptualization, Formal analysis, Investigation, Methodology, Project administration, Visualization, Writing—original draft, Writing—review & editing.
